# NFATc1 induction by an intronic enhancer restricts NKT γδ cell formation

**DOI:** 10.1016/j.isci.2023.106234

**Published:** 2023-02-19

**Authors:** Sabrina Giampaolo, Cristina M. Chiarolla, Konrad Knöpper, Martin Vaeth, Matthias Klein, Azeem Muhammad, Tobias Bopp, Friederike Berberich-Siebelt, Amiya K. Patra, Edgar Serfling, Stefan Klein-Hessling

**Affiliations:** 1 Institute of Pathology, Julius Maximilians University Würzburg, Josef-Schneider-Strasse 2, 97080 Würzburg, Germany; 2 Würzburg Institute of Systems Immunology, Max Planck Research Group at the Julius-Maximilians-University Würzburg, Versbacher Strasse 9, 97078 Würzburg, Germany; 3 Institute for Immunology, University Medical Center, University of Mainz, Langenbeckstraße 1, 55131 Mainz, Germany; 4 Peninsula Medical School, University of Plymouth, The John Bull Building, Plymouth Science Park, Research Way, Plymouth PL6 8BU, UK; 5 Comprehensive Cancer Center Mainfranken, Würzburg, Germany

**Keywords:** molecular biology, Immunology, developmental biology

## Abstract

In thymus, the ablation of T cell receptor (TCR)-activated transcription factor NFATc1 or its inducible isoforms during the double-negative (DN) stages of thymocyte development leads to a marked increase in γδ thymocytes whereas the development of αβ thymocytes remains mostly unaffected. These γδ thymocytes are characterized by the upregulation of the promyelocytic leukemia zinc-finger factor (PLZF), the “master regulator” of natural killer T (NKT) cell development, and the acquisition of an NKT γδ cell phenotype with higher cell survival rates. The suppressive function of NFATc1 in NKT γδ cell formation critically depends on the remote enhancer E2, which is essential for the inducible expression of NFATc1 directed by its distal promoter P1. Thus, the enhancer deciphers a strong γδ TCR signal into the expression of inducible NFATc1 isoforms resulting in high levels of NFATc1 protein that are essential to control the numbers of NKT γδ cells.

## Highlights

• Ablation of NFATc1 leads to the expansion of PLZF^+^ NK γδ thymocytes
• High levels of NFATc1 in γδ thymocytes depend on the activity of the E2 enhancer
• The inducible form NFATc1/α enforces cell death of NK γδ thymocytes

## Graphical abstract

**Figure:**
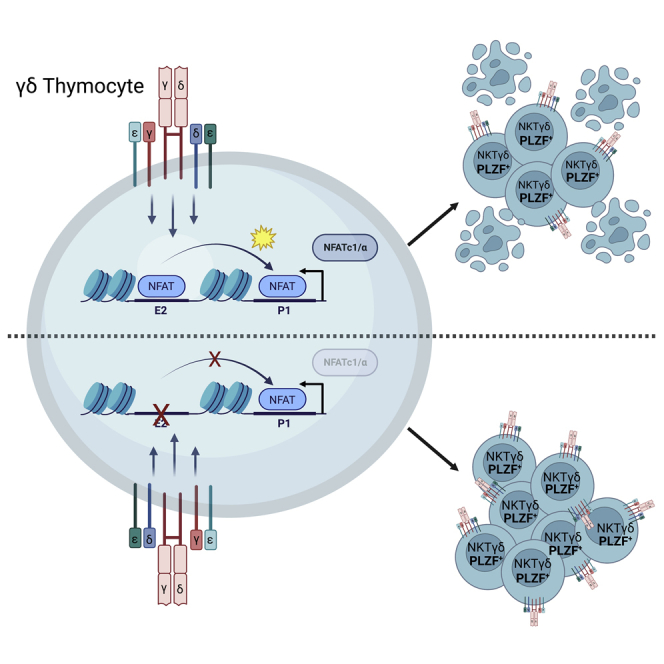

## Introduction

The thymus is a primary lymphoid organ and the site where lymphoid progenitors, moving from fetal liver or bone marrow, differentiate into T cells. In adult mice, common lymphoid progenitors (CLPs) from the bone marrow (BM) enter the thymus from the blood at the cortico-medullary junction, migrate into the organ, and differentiate. Along with conventional αβ T cells, so-called unconventional T cells including γδ T cells, invariant (iNKT) and diverse natural killer T (NKT) cells (dNKT), NKT γδ cells, and the CD8αα^+^ TCRαβ intraepithelial lymphocyte precursors (IELPs) develop in the thymus.^1^^,^^2^

Thymocyte development can be classified into different stages based on the expression of surface markers, including the co-receptors CD4 and CD8, and the corresponding T cell receptors (TCRs). During the first stages of T cell lineage development, thymocytes lack both CD4 and CD8 co-receptors expression and are therefore named double-negative (DN). Based on the surface expression of CD44 and CD25, DN thymocytes can be subdivided into four distinct subpopulations: ETP-DN1 (CD44^+^ CD25^−^), DN2 (CD44^+^ CD25^+^), DN3 (CD44^−^ CD25^+^), and DN4 (CD44^−^ CD25^−^) cells.^3^^,^^4^ At the point of transition from DN2 to DN3 stages, expression of the *Rag1* and *Rag2* genes (recombination activating genes 1 and 2) occurs. *Tcrg*, *Tcrd,* and *Tcrb* loci are rearranged in thymocytes thus leading to the expression of the TCRγδ or the β-chain of the TCR.^4^ The TCRβ chain pairs with a surrogate α chain (pre-Tα) to form the pre-T cell receptor (pre-TCR). This step represents the time point of αβ and γδ bifurcation.^5^

γδ T cells are a unique and well-conserved population of lymphoid cells. Different from αβ T and B cells, γδ T cells display characteristics of cells of both the innate and adaptive immune system. In mice, they account for 4% of T cells in the thymus and secondary lymphoid organs. It is still controversial which signals induce the common DN progenitors to follow one or the other lineage. Current studies support the TCR-dependent strength model. Similarly, it has been shown that NKT cells, a population belonging to the unconventional innate-like group, require a strong TCR signal for selection.^6^^,^^7^^,^^8^

NKT cells are a subset of innate-like T cells, which share innate characteristics of NK cells and adaptive functions like T lymphocytes, therefore owning the ability to bridge innate and adaptive immunity.^9^ They undergo a selection in the thymus and recognize various lipid antigens presented on CD1d molecules.^10^ With the other innate-like cells, they share effector signatures, transcription factors, and surface markers.^10^^,^^11^^,^^12^^,^^13^ In mice, while most of them express a αβ TCR, there is also a population of NKT cells expressing NK1.1 and Vγ1.^2^^,^^14^

The promyelocytic leukemia zinc-finger (PLZF) transcription factor is essential for developing and acquiring the phenotype of NKT cells.^15^^,^^16^ PLZF is induced by strong TCR signals in the NKT cells.^16^^,^^17^ Likewise, PLZF is the main regulator of a cell population that resembles NKT cells, which express preferentially the Vγ1.1/Vδ6.3 TCR segments, produce the same cytokines, and share similar surface markers. They were designated as NKT γδ cells^18^^,^^19^^,^^20^^,^^21^ or recently Tγδ2.^13^

The family of nuclear factor of activated T cell (NFAT) of transcription factors consists of five members: NFATc1 (NFAT2), NFATc2 (NFAT1), NFATc3 (NFAT4), NFATc4 (NFAT3), and NFAT5.^22^^,^^23^ Six isoforms with partly overlapping functions have been described for NFATc1, a prominent NFAT factor in nuclei of activated T cells.^24^^,^^25^ They are generated by the differential usage of two promoters, two poly-A sites, pA1 and pA2, and alternative splicing events. Inducible NFATc1/α isoforms are generated under the control of the TCR-dependent distal promoter P1, while constitutive NFATc1/β isoforms are directed by the proximal promoter P2.^26^^,^^27^ An important regulatory element that contributes to the full transcriptional activity of the P1 promoter is the enhancer 2 (E2), located within the intron 10 of the *Nfatc1* gene.^28^ While the role of NFATc1 in peripheral T cell function and differentiation has been well established, less attention was paid to the role of NFATc1 during thymocyte development. However, we previously observed strong expression of NFATc1 in the DN population, as compared to double positive (DP) and single positive (SP) thymocytes. Among the DN populations, the pre-TCR-negative population of DN3 thymocytes showed the highest NFATc1 expression level.^29^^,^^30^

In this study, we investigated the role of NFATc1 during the DN stages of early thymocyte development further. While in thymi of both *Rag1Cre-Nfatc1*^*fl/fl*^ and *Rag1Cre-E2*^*fl/fl*^ mice, in which the synthesis of all or inducible NFATc1 proteins is abolished, a moderate decrease in overall thymocyte numbers was observed, we detected a marked expansion in γδ thymocytes. Those γδ thymocytes exhibited a strong increase in the expression of PLZF, the ”master regulator” of NKT cells, and the acquisition of an NKT γδ phenotype. These data suggest that, due to the induction by γδ TCR activation in DN3 thymocytes, high NFATc1 levels increase the susceptibility for apoptosis in NKT γδ cells and thereby control their expansion.

## Results

### Lack of NFATc1 activity leads to an increased number of γδ thymocytes

**Figure 1:**
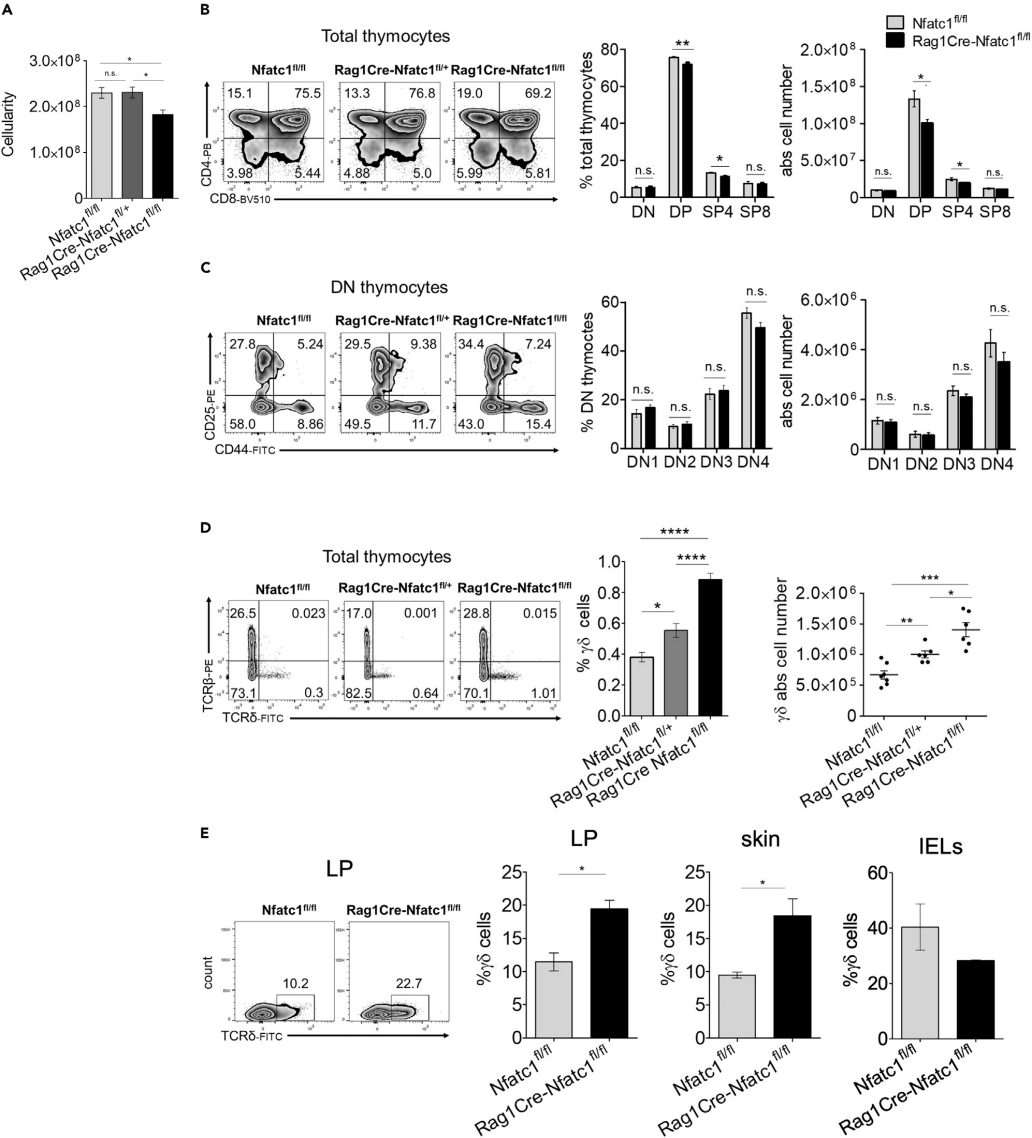
NFATc1 ablation in DN thymocytes leads to increased numbers of **γδ** T cells (A) Thymic cellularity in the *Nfatc1*^*fl/fl*^, *Rag1Cre-Nfatc1*^*fl/+*^, and *Rag1Cre-Nfatc1*^*fl/fl*^ mice. (B) Flow cytometry of total thymocytes from *Nfatc1*^*fl/fl*^, *Rag1Cre-Nfatc1*^*fl/+*^, and *Rag1Cre-Nfatc1*^*fl/fl*^ mice. Cells were stained with anti-CD4 and anti-CD8. Results of one representative experiment (left), the percentages (mid), and absolute cell numbers (right) of DN, DP, and SP (CD4^+^ and CD8^+^) thymocytes from *Nfatc1*^*fl/fl*^ and *Rag1Cre-Nfatc1*^*fl/fl*^ mice are shown. (C) Flow cytometry of total thymocytes from *Nfatc1*^*fl/fl*^, *Rag1Cre-Nfatc1*^*fl/+*^, and *Rag1Cre-Nfatc1*^*fl/fl*^ mice gated on DN thymocytes. Cells were stained with anti-CD25 and anti-CD44. Results of one representative experiment (left), the percentages (mid), and absolute cell numbers (right) of the DN subpopulations from *Nfatc1*^*fl/fl*^ and *Rag1Cre-Nfatc1*^*fl/fl*^ mice are shown. (D) Thymocyte flow cytometry from *Nfatc1*^*fl/fl*^, *Rag1Cre-Nfatc1*^*fl/+*^, and *Rag1Cre-Nfatc1*^*fl/fl*^ mice. Total thymocytes were stained with anti-TCRδ and anti-TCRβ and gated on living cells. The percentages and absolute numbers of γδ T cells in thymi of mice are shown in the mid and right panels. Each dot represents one mouse. (E) Representative flow cytometry plot of the accumulation of γδ T cells in the intestine’s lamina propria (LP). Right, percentages of γδ T cells in LP, skin, and IELs from *Nfatc1*^*fl/fl*^ and *Rag1Cre-Nfatc1*^*fl/fl*^ mice. Data from six independent assays are compiled and shown as mean ± SEM. The statistical significance was determined by unpaired student’s t-tests. ∗p value <0.05, ∗∗p value <0.005, ∗∗∗p value <0.001, ∗∗∗∗p value <0.0001, n.s. not significant. See also Figure S1.

In our previous study, ablation of NFATc1 in hematopoietic T cell progenitors led to the shrinking of lymphatic organs, a dramatic decrease in thymic cellularity, and a block of thymocyte development at the DN1 stage.^30^ To check whether the inactivation of the *Nfatc1* gene in early DN thymocytes exerts a similarly dramatic effect on overall thymic development, we investigated thymocyte development in *Rag1Cre-Nfatc1*^*fl/fl*^ mice. In those mice, the Cre recombinase expressed under the control of the *Rag1* locus causes the inactivation of the target gene very efficiently at the DN2 to DN3 stages.^31^ This resulted in a strong decrease in *Nfatc1* RNA levels both in DN and in DP and SP thymocytes (Figure S1A). However, the loss of NFATc1 expression in early thymocytes resulted only in a mild reduction of overall thymic cellularity in *Rag1Cre-Nfatc1*^*fl/fl*^ mice (Figure 1A). While a subtle decrease in DP and CD4^+^ single-positive thymocytes was detected, almost no difference was observed in the distribution of subpopulations of DN thymocytes between *Nfatc1*^*fl/fl*^ control and *Rag1Cre-Nfatc1*^*fl/fl*^ mice (Figures 1B and 1C). When we studied the lineage distribution of thymocytes from *Nfatc1*^*fl/fl*^, *Rag1Cre-Nfatc1*^*fl/+*^, and *Rag1Cre-Nfatc1*^*fl/fl*^ mice by flow cytometry, a slight but non-significant decrease was observed in the percentage and absolute numbers of TCRαβ thymocytes in total population (Figures 1D and S1B). However, these assays revealed a conspicuous NFATc1 dose-dependent increase in the percentage of γδ thymocytes between those mouse lines. Absolute numbers of γδ thymocytes in *Rag1Cre-Nfatc1*^*fl/fl*^ mice increased more than 2-fold compared to control mice (Figure 1D). These results prompted us to further analyze γδ lineage development. We performed intracellular staining of TCRβ and TCRδ chains. The data of flow cytometry assays revealed again a marked increase in TCRδ^+^ cells in the populations of total and DN thymocytes from *Rag1Cre-Nfatc1*^*fl/fl*^ mice, and the proportion of TCRβ^+^ cells was comparable both in total and in DN thymocytes. It is Noteworthy that we also observed a significant increase in the population of NFATc1-deficient thymocytes expressing both the intracellular TCRβ and TCRδ chains (0.18% in total *Nfatc1*^*fl/fl*^ vs 0.35% in *Rag1Cre-Nfatc1*^*fl/fl*^ and 1.48% in DN *Nfatc1*^*fl/fl*^ vs 3.05% in *Rag1Cre-Nfatc1*^*fl/fl*^) (Figures S1C and S1D). We also confirmed by RT-PCR analysis that the defect in *Nfatc1* expression was not compensated by changes in *Nfatc2* and *Nfatc3* expression (Figure S1E). Taken together, these results suggest that during the DN stages of thymocyte development, NFATc1 plays a relevant role in the regulation of γδ T cell development.

To exclude the possibility that γδ thymocytes accumulate in the thymus, we analyzed the presence of γδ T cells in some homing tissues, such as in the intestine and skin. We observed a heightened percentage of γδ T cells among the lymphocytes from lamina propria (LP) and skin of *Rag1Cre-Nfatc1*^*fl/fl*^ mice, but not within the intraepithelial lymphocyte (IEL) population (Figures 1E and S1F). Therefore, the absence of NFATc1 leads to an increase in γδ T cells in thymus and peripheral organs. This suggests that NFATc1 plays a role in the phenotype specification of γδ subpopulations during thymocyte development.

### The absence of NFATc1 leads to a change in γδ thymocyte differentiation

**Figure 2:**
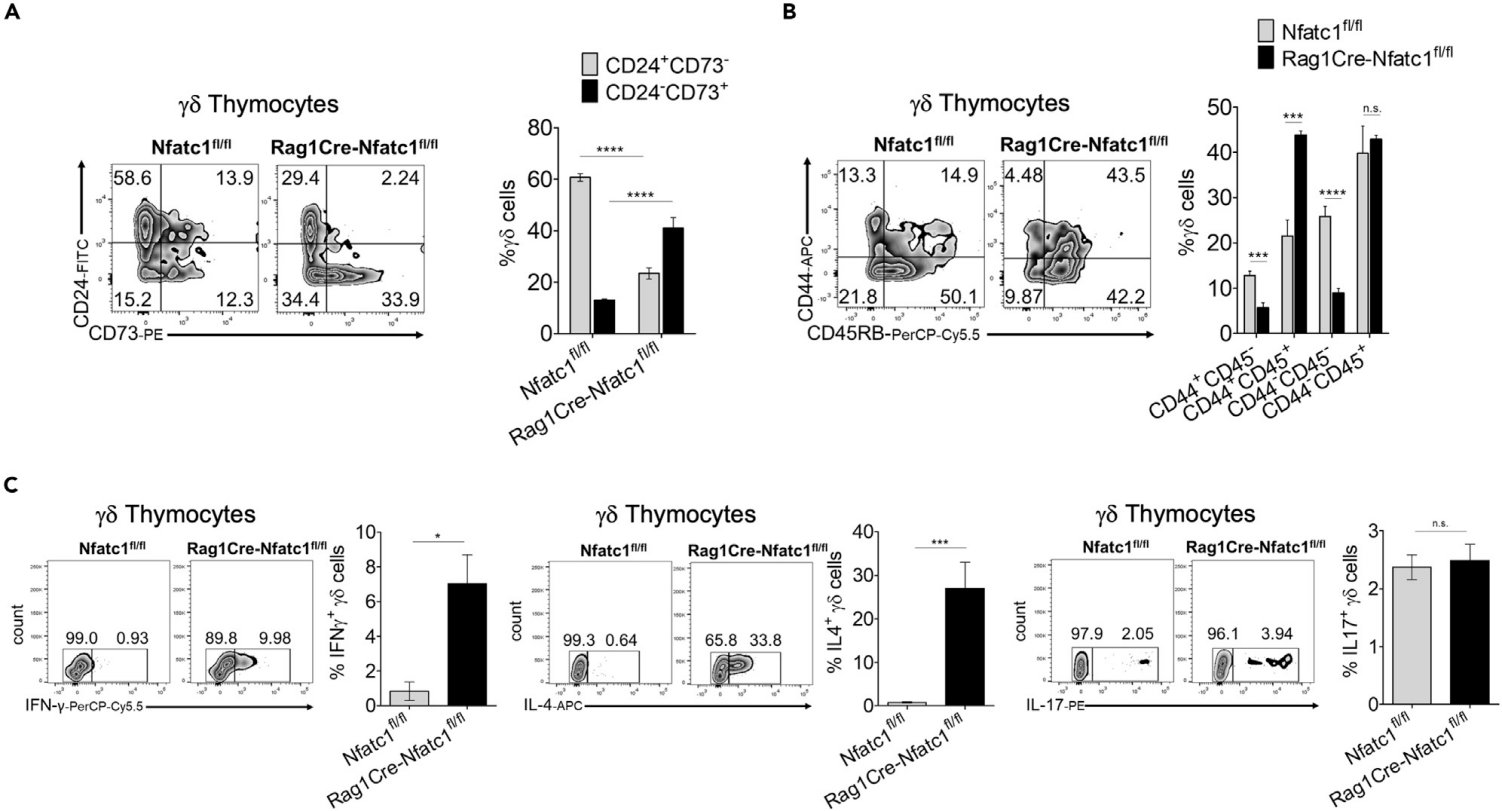
NFATc1 absence affects γδ thymocyte differentiation (A) Flow cytometry analysis of γδ thymocytes from *Nfatc1*^*fl/fl*^ and *RagCre-Nfatc1*^*fl/fl*^ mice upon staining with anti-CD24 and anti-CD73. Right, quantification of results of at least four assays. (B) Flow cytometry of γδ thymocytes from *Nfatc1*^*fl/fl*^ and *RagCre-Nfatc1*^*fl/fl*^ mice upon staining with anti-CD44 and anti-CD45RB. Right, percentages of TCRδ^+^ thymocytes expressing CD44 and CD45RB surface markers. (C) Flow cytometry of γδ thymocytes producing IFN-γ, IL-4, and IL-17. Each flow cytometry plot is associated with its corresponding percentages of γδ thymocytes. Data representative for at least three independent experiments with four mice from every genotype are shown as mean ± SEM. The statistical significance was determined by unpaired student’s t-tests. ∗p value <0.05, ∗∗∗p value <0.001, ∗∗∗∗p value <0.000, n.s. not significant.

We next characterized the γδ thymocyte population in *Rag1Cre-Nfatc1*^*fl/fl*^ mice for maturation surface markers and functional identity. Among the surface markers that characterize the developmental stages of γδ T cells during thymocyte development, we measured the expression of CD24 and CD73 as indicators for cell maturation and commitment.^14^ γδ thymocytes move along from more immature CD24^+^CD73^−^ cells to mature CD24^−^CD73^+^ stages,^32^ where CD73 expression corresponds to a definitive commitment toward the γδ lineage.^33^ Flow cytometry showed an increase in the percentage of γδ thymocytes with no or lower expression of CD24 and an increase in CD73 surface marker expression on *Rag1Cre-Nfatc1*^*fl/fl*^ γδ thymocytes (Figure 2A). These data suggest that NFATc1-deficient γδ thymocytes are fully committed to the γδ cell lineage.

Previous research has shown that the expression of CD44 and CD45RB surface markers on γδ thymocytes correlates with a functional fate in the thymus.^32^ Our analysis of surface markers showed a strong increase in CD44^+^ CD45RB^+^ cells in γδ thymocytes lacking NFATc1 (Figure 2B).

Next, we investigated the cytokine production capacity of γδ thymocytes from *Rag1Cre-Nfatc1*^*fl/fl*^ mice. After induction with phorbol 12-myristate 13-acetate (PMA) and ionomycin for 5 h, an increased percentage of *Rag1Cre-Nfatc1*^*fl/fl*^ γδ thymocytes produced interferon (IFN)-γ (∼10% IFN-γ^+^ in *Rag1Cre-Nfatc1*^*fl/fl*^ mice vs ∼1% IFN-γ^+^ in *Nfatc1*^*fl/fl*^ mice) and, to a greater extent, interleukin-4 (IL-4) (∼30% IL-4^+^ in *Rag1Cre-Nfatc1*^*fl/fl*^ mice vs ∼0.6% IL-4^+^ in *Nfatc1*^*fl/fl*^ mice) (Figure 2C), while no change in IL-17-producing thymocytes was detected. Therefore, in the absence of NFATc1, more γδ thymocytes differentiate into IFN-γ and especially IL-4-producing cells.

### NFATc1-deficient γδ T cells acquire an NKT γδ cell phenotype

**Figure 3:**
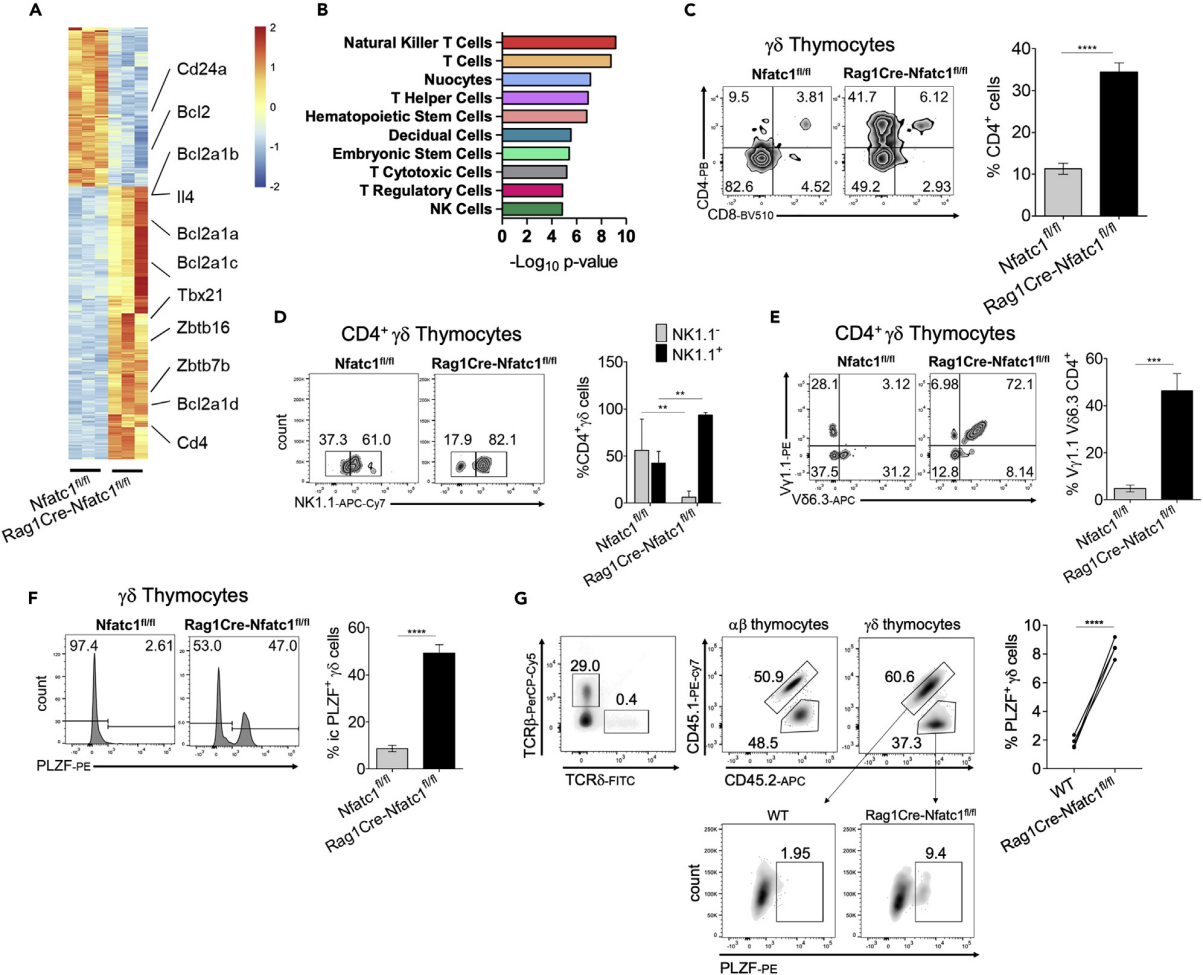
γδ thymocytes from *Rag1Cre-Nfatc1*^*fl/fl*^ mice exhibit an NKT γδ phenotype (A) Differently expressed genes from freshly isolated *Rag1Cre-Nfatc1*^*fl/fl*^ and *Nfatc1*^*fl/fl*^ γδ thymocytes. (B) Cell type identification analysis of RNA-seq data of *Nfatc1*^*fl/fl*^ and *RagCre-Nfatc1*^*fl/fl*^ γδ thymocytes shows the dominance of NKT cell phenotype. (C) Flow cytometry of γδ thymocytes from *Nfatc1*^*fl/fl*^ and *RagCre-Nfatc1*^*fl/fl*^ mice upon staining with anti-CD4 and anti-CD8. Right, percentages of CD4^+^ γδ thymocytes. (D) Flow cytometry of thymocytes from *Nfatc1*^*fl/fl*^ and *RagCre-Nfatc1*^*fl/fl*^ mice gated for CD4^+^ TCRδ^+^ cells upon staining with anti-NK1.1. Right, percentages of CD4^+^ TCRδ^+^ thymocytes expressing the NK1.1 surface marker. (E) Flow cytometry of total thymocytes from *Nfatc1*^*fl/fl*^*and RagCre-Nfatc1*^*fl/fl*^ mice gated for CD4^+^ TCRδ^+^ cells upon staining with anti-Vγ1.1 and anti-Vδ6.3. Right, percentages of CD4^+^ TCRδ^+^ thymocytes expressing the Vγ1.1^+^ and Vδ6.3^+^ gene segments. (F) Number of γδ thymocytes from *Nfatc1*^*fl/fl*^ and *RagCre-Nfatc1*^*fl/fl*^ mice expressing the transcription factor PLZF analyzed by flow cytometry (left) and percentages of γδ thymocytes expressing PLZF (right). (G) (Above) Flow cytometry analysis of PLZF^+^ γδ thymocytes from WT (CD45.1^+^CD45.2^+^) and *Rag1Cre-Nfatc1*^*f/fl*^ (CD45.2^+^) mouse bone marrow chimera. Total thymocytes are gated for TCRδ and TCRβ expression. The percentages of γδ PLZF^+^ thymocytes is calculated from the WT and the *Rag1Cre-Nfatc1*^*f/fl*^ γδ thymocyte compartments. Data are representative of at least two independent experiments with four mice from every genotype and are shown as mean ± SEM. The statistical significance was determined by unpaired student’s t-tests. ∗∗p value <0.005, ∗∗∗p value <0.001, ∗∗∗∗p value <0.000. See also Figure S2.

To check if the ablation of NFATc1 in DN thymocytes leads to an altered differentiation in those γδ thymocytes, we performed RNA sequencing (RNA-seq) assays using γδ thymocytes isolated from *Rag1Cre-Nfatc1*^*fl/fl*^ and control mice (Figure 3A). We observed downregulation of the *Cd24a* gene, which was obvious from flow cytometry already (Figure 2A)*.* The RNA-seq data also confirmed the upregulation of *Il4* gene expression (Figure 2C). Strikingly, unbiased cell-type identification using the transcriptome from isolated NFATc1-deficient γδ thymocytes identified a signature associated with NKT cells (Figure 3B).

As a part of the NKT population retains the expression of CD4 during development^10^^,^^12^^,^^34^ and several studies showed CD4 expression in an expanded population of NKT γδ cells in mouse models with TCR signaling defects,^16^^,^^35^ we measured CD4 expression on γδ thymocytes. Flow cytometry revealed that in *Rag1Cre-Nfatc1*^*fl/fl*^ mice 4-fold more γδ thymocytes expressed the CD4 co-receptor compared to those in *Nfatc1*^*fl/fl*^ mice (41.7% *Rag1Cre-Nfatc1*^*fl/fl*^ and 9.05% *Nfatc1*^*fl/fl*^) (Figure 3C). Flow cytometry of the “classical” NK1.1 surface marker revealed a significant increase in its expression on NFATc1^−/−^ γδ thymocytes (Figure 3D). In addition, we observed an increase in CD4^+^ γδ thymocytes expressing Vγ1.1 and Vδ6.3 gene segments (TCRV), typical for NKT γδ cells, in mice bearing NFATc1-deficient thymocytes (Figure 3E). However, analysis using Cd1d tetramers in thymi from *Rag1Cre-Nfatc1*^*fl/fl*^ and control mice revealed no differences in the conventional NKT αβ population (Figure S2A).

Among several differentially transcribed transcription factors, we recognized that *Zbtb16,* the gene which encodes the transcription factor PLZF, was strongly upregulated in the absence of NFATc1 (Figure 3A). PLZF has been described as a “master regulator” of NKT and NKT γδ development.^15^^,^^18^ We also observed a similar strong upregulation of *Zbtb16* mRNA using *Rag1Cre-Nfatc1*^*fl/fl*^ DN thymocytes in RT-PCR assays (Figure S2B). Flow cytometry showed a much higher number of γδ thymocytes from *Rag1Cre-Nfatc1*^*fl/fl*^ mice compared to *Nfatc1*^*fl/fl*^ control mice (47% vs 2.6%) expressing PLZF factor (Figure 3F). This increase in PLZF^+^ cells is most prominent in the CD4^+^ γδ subpopulation of *Rag1Cre-Nfatc1*^*fl/fl*^ thymocytes (Figure S2C). To prove that CD4^+^ γδ thymocyte expansion is led by an intrinsic mechanism, we analyzed the thymocytes from bone marrow chimeric mice. CD45.1^+^ *Rag2*^*−/−*^ *γc*^*−/−*^ recipient mice were reconstituted with CD45.1^+^CD45.2^+^ wild-type (WT) and CD45.2^+^ *Rag1Cre-Nfatc1*^*fl/fl*^ bone marrow cells in a ratio of 1:1. While both types contribute equally to the generation of all thymic populations (Figure S2D), the percentage of PLZF^+^ thymocytes was significantly increased in the NFATc1-deficient compartment of γδ thymocytes compared to wild type (Figure 3G).

### Reduced cell death in NFATc1-deficient NKT γδ thymocytes

**Figure 4:**
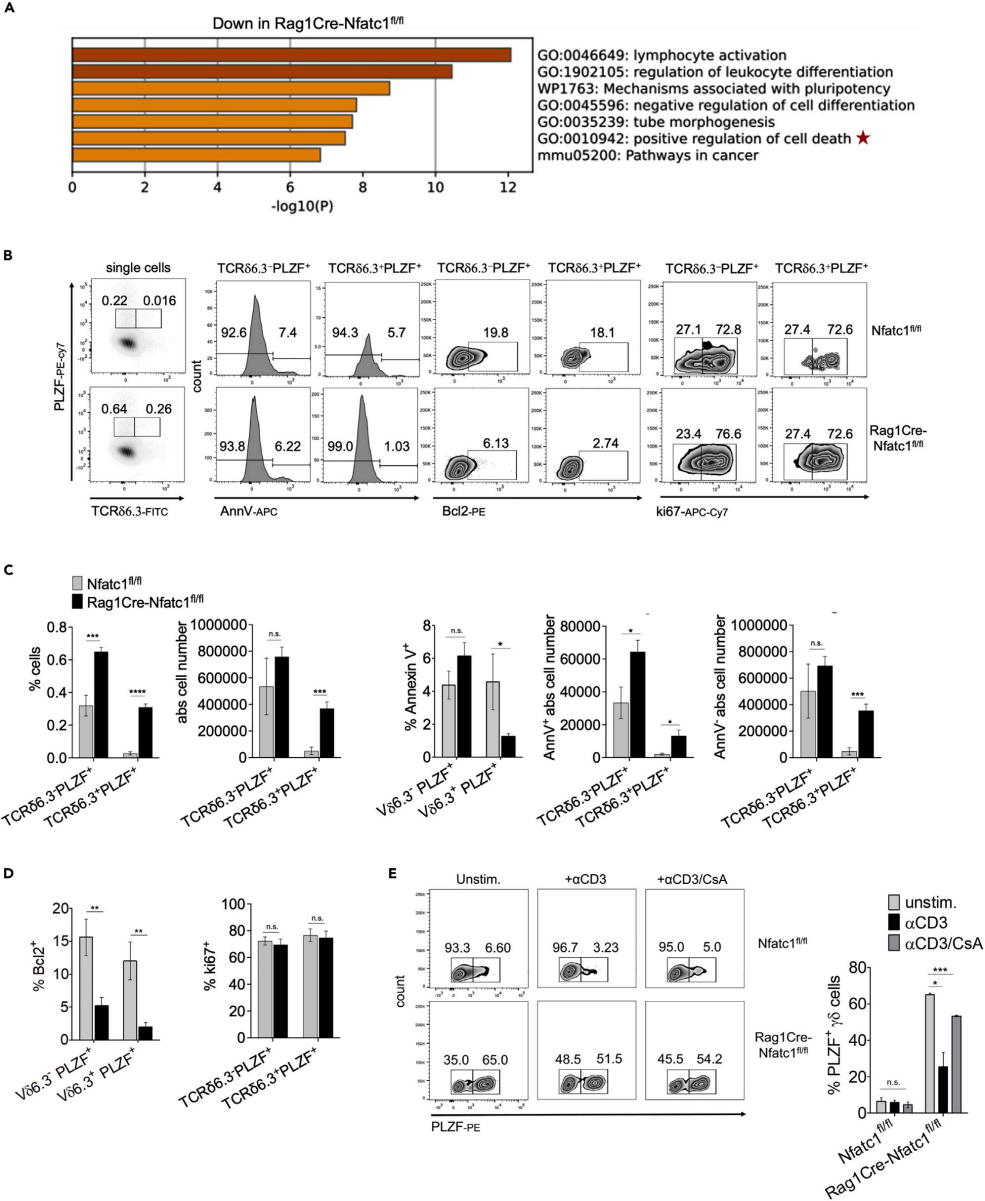
The absence of NFATc1 confers a better survival rate to PLZF^+^ TCRδ6.3^+^ thymocytes from *Rag1Cre-Nfatc1*^*fl/fl*^ mice (A) Gene Ontology (GO) analysis of the genes differentially expressed in *Rag1Cre-Nfatc1*^*fl/fl*^ compared to control thymocytes. The red star indicates the enrichment of genes involved in “positive regulation of cell death” pathway downregulated in NFATc1-deficient γδ thymocytes. (B) Annexin V (left), Bcl2 (mid), and ki67 (right) staining of *Rag1Cre-Nfatc1*^*fl/fl*^ and control thymocytes gated for the expression of PLZF and TCRVδ6.3 gene segment. (C) Corresponding percentages and absolute cell numbers of PLZF^+^ TCRVδ6.3^-^ and TCRVδ6.3^+^ thymocyte populations analyzed by flow cytometry as reported in (b). (Left) percentages and absolute cell numbers of PLZF^+^ TCRVδ6.3^-^ and TCRVδ6.3^+^ subpopulations, and (right) percentages of annexin V^+^ and absolute numbers of annexin V^+^ and annexin V^−^ PLZF^+^ TCRVδ6.3^-^ and TCRVδ6.3^+^ thymocytes. (D) Percentages of Bcl2-expressing PLZF^+^ TCRVδ6.3^-^ and TCRVδ6.3^+^ thymocytes (left) and percentages of PLZF^+^ TCRVδ6.3^-^ and TCRVδ6.3^+^ positively stained for ki67 protein (right). (E) Cell numbers of *Rag1Cre-Nfatc1*^*fl/fl*^ and control γδ thymocytes expressing PLZF stimulated with αCD3, or αCD3 and CsA for 24 h or left unstimulated. (Right) Percentages of γδ thymocytes expressing PLZF. Data are representative of at least two independent experiments with four mice from every genotype and are shown as mean ± SEM. The statistical significance was determined by unpaired student’s t-tests. ∗p value <0.05, ∗∗p value <0.005, ∗∗∗p value <0.001, n.s. not significant. See also Figure S3.

Pathway analysis of our RNA-seq data revealed in *Rag1Cre-Nfatc1*^*fl/fl*^ γδ thymocytes a downregulation of genes involved in the positive control of cell death, pointing out a function of NFATc1 in controlling γδ thymocyte survival (Figure 4A). To address the cause of NKT γδ thymocyte increase in *Rag1Cre-Nfat/c1*^*fl/fl*^ thymi, we measured the apoptosis levels of different thymocyte subsets by annexin V staining. When PLZF^+^ thymocytes were gated for TCR Vδ6.3^-^ and Vδ6.3^+^ populations, we observed reduced annexin V staining in PLZF^+^ Vδ6.3^+^ NKT γδ cells upon NFATc1 ablation leading to a strong increase in the absolute number of annexin V-negative NKT γδ thymocytes. Nevertheless, control PLZF^+^ Vδ6.3^-^ NKT cells were not affected (Figures 4B and 4C). Similarly, when Vγ1.1^+^ γδ cells in *Rag1Cre-Nfatc1*^*fl/fl*^ thymocytes were gated for PLZF^−^ and PLZF^+^ expression, the PLZF^+^ Vγ1.1^+^ NKT γδ population showed reduced percentage and absolute cell numbers of annexin V-positive cells in those mice (Figures S3A and S3B).

This increased survival rate is not due to a difference in the expression of the anti-apoptotic family member Bcl-2 since we observed lower frequencies of Bcl-2 positive cells in all analyzed Vδ6.3, Vγ1.1, and PLZF-expressing subpopulations of *Nfatc1*-deficient thymocytes (Figures 4B, 4D, S3A, and S3C). RNA-seq assays revealed a switch in the expression of the anti-apoptotic members of the Bcl2 family; while *Bcl2* transcripts decreased, an upregulation of the *Bcl2a1* genes was evident (Figure 3A). To corroborate this conclusion, we measured the expression of anti-apoptotic Bcl2 family members by RT-PCR. Transcriptional analysis showed the strong upregulation of *Bcl2a1a* and, to a lower extent, of *Bcl2l1* (coding for Bcl-XL) in isolated γδ thymocytes from *Rag1Cre-Nfatc1*^*fl/fl*^ mice, whereas the expression of the *Bcl2* gene was downregulated (Figure S3D). To evaluate the possibility that the lack of NFATc1 could confer a proliferation advantage of *Rag1Cre-Nfatc1*^*fl/fl*^ γδ compared to WT thymocytes, we performed a proliferation analysis of γδ thymocyte subpopulations by Ki67 protein staining. Our data revealed no significant difference within the TCRVδ6.3^+^ and TCRVδ6.3^-^ expressing PLZF factor and TCRVγ1.1 PLZF^–^ and PLZF^+^ subpopulations (Figures 4B, 4D, S3A, and S3C).

To unravel the molecular mechanisms that support the survival of NKT γδ in *Nfatc1*^−/−^ thymocytes, we stimulated thymocytes *in vitro* by αCD3 for 24 h and detected fewer γδ thymocytes from *Rag1Cre-Nfatc1*^*fl/fl*^ mice with PLZF expression, compared to untreated cells. The addition of cyclosporin A (CsA), a specific inhibitor of Ca^++^-dependent phosphatase calcineurin, to αCD3-treated cells led to a “rescue” in the number of γδ thymocytes, which expressed PLZF (Figure 4E). Taken together, our data show that the ablation of NFATc1 supports the survival of γδ thymocytes that express PLZF while strong TCR stimulation *in vitro* by αCD3 reduces their number.

### Strong NFATc1 expression in γδ thymocytes

**Figure 5:**
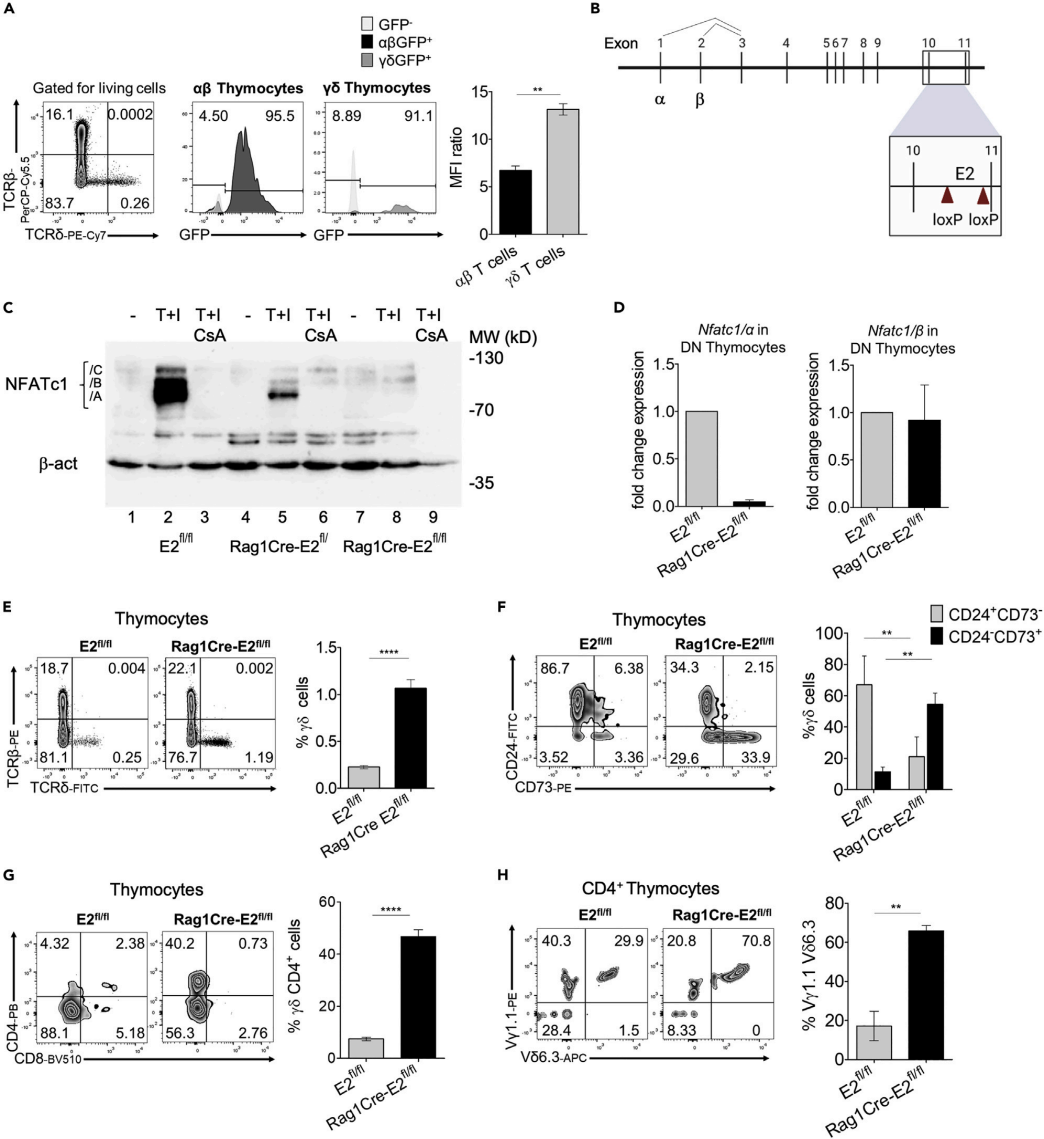
Specific depletion of inducible NFATc1/α isoforms leads to the occurrence of NKT γδ cells in thymus (A) NFATc1 expression in thymocytes from *Nfatc1-eGfp-Bac* reporter mice. Total thymocytes were stained with anti-TCRβ and anti-TCRδ. The GFP reporter signal was measured by flow cytometry in each gated population. Left, percentages of αβ vs γδ lymphocytes-expressing GFP from *Nfatc1-eGfp-Bac* mice, and right, MFI ratio in αβ and γδ total thymocytes. (B) Scheme of the *Nfatc1* gene with two *loxP* sites flanking the remote enhancer E2 in intron 10. The coding exons for the α- and β-isoforms are marked. (C) NFATc1 protein levels in thymocytes from *E2*^*fl/fl*^, *RagCre-E2*^*fl/+*^, and *RagCre-E2*^*fl/fl*^ mice were measured by western blots with β-actin as a loading control. Total thymocytes were induced with TPA and ionomycin (T + I) alone, without or with the addition of cyclosporin A (CsA) for 4 h or left untreated (−). The position of the isoforms A, B, and C is indicated. (D) Quantification of the inducible *Nfatc1/α* (left) and the constitutive *Nfatc1/β* transcripts in DN thymocytes from *E2*^*fl/fl*^ or *RagCre-E2*^*fl/fl*^ mice. Transcript levels were normalized to the housekeeping gene *B2m* and shown as fold change in expression relative to control. (E) Flow cytometry of thymocytes from *E2*^*fl/fl*^ and *RagCre-E2*^*fl/fl*^ mice. Total thymocytes were stained with anti-TCRδ and anti-TCRβ. The percentages of each population are indicated in the respective quadrant. Gated on living cells. Right, percentages of γδ T cells in thymi. (F) Flow cytometry of γδ thymocytes from *E2*^*fl/fl*^ and *RagCre-E2*^*fl/fl*^ mice upon staining with anti-CD24 and anti-CD73. Right, percentages of γδ cells stained for CD24 and CD73 markers. (G) Flow cytometry of thymocytes from *E2*^*fl/fl*^ and *RagCre-E2*^*fl/fl*^ mice stained with anti-CD4 and anti-CD8 and gated for total γδ T cells. Right, percentages of CD4^+^ γδ T cells in total γδ thymocytes. (H) Total thymocytes from *E2*^*fl/fl*^*and RagCre-E2*^*fl/fl*^ mice gated for CD4^+^ TCRδ^+^ cells upon staining with anti-Vγ1.1 and anti-Vδ6.3. Right, percentages of CD4^+^ TCRδ^+^ thymocytes expressing Vγ1.1 and Vδ6.3 chains. Data representative for at least three independent experiments with four mice from every genotype are shown as mean ± SEM. The statistical significance was determined by unpaired student’s t-tests. ∗∗p value <0.005, ∗∗∗∗p value <0.0001. See also Figures S4 and S5.

The increase in γδ thymocytes upon NFATc1 ablation prompted us to investigate NFATc1 expression in αβ and γδ thymocytes. Using *Nfatc1-eGfp-Bac* reporter mice that express an EGFP indicator gene under the control of the *Nfatc1* locus,^29^^,^^36^ we detected similar percentage of γδ and αβ thymocytes expressing GFP (∼93.3%). However, the NFATc1-mediated GFP expression was stronger in γδ compared to αβ thymocytes, as indicated in the MFI ratio level between the two populations (Figure 5A). In gated DN thymocytes, we observed a significant difference in NFATc1 expression between γδ and αβ thymocytes but to a lesser extent compared to that in total thymocytes. (Figure S4A). Similar results were obtained for γδ T cells from lymph nodes and spleen. There, again, NFATc1 was more strongly expressed in γδ T cells compared to αβ cells (Figure S4B). The same analysis revealed that the few CD4^+^ γδ thymocytes expressed a higher level of NFATc1 compared to αβ thymocytes (Figure S4C).

### *Nfatc1* enhancer E2 is essential for the suppression of NKT γδ cell development

We showed previously that the expression of pre-TCR in DN3 thymocytes is closely linked to the transcriptional induction of NFATc1 and the generation of NFATc1/α isoforms in these cells.^28^ To test whether this NFATc1 induction in DN3 thymocytes suppresses γδ lineage development, we investigated thymocyte development in *Rag1Cre-E2*^*fl/fl*^ mice, a newly generated mouse line, in which the remote enhancer E2^28^ is deleted (Figure 5B). This leads to specific inhibition of NFATc1 induction by preventing the expression of NFATc1/α but not of the constitutive NFATc1/β isoforms (Figures 5C and 5D). As for the complete ablation of all NFATc1 proteins in *Rag1Cre-Nfatc1*^*fl/fl*^ mice, we observed a moderate decrease in the number of total thymocytes (Figure S5A) but a marked, 7-fold increase in the percentage of γδ thymocytes in *Rag1Cre-E2*^fl/fl^ mice compared to control littermates (1.17% *Rag1Cre-E2*^*fl/fl*^ vs 0.16% *E2*^*fl/fl*^ control) (Figure 5E). As confirmed by flow cytometry, this increase in γδ thymocytes was not paralleled by a significant decrease in αβ thymocytes (Figure S5B).

Similar to *Rag1Cre-Nfatc1*^*fl/fl*^ mice, NFATc1/α-deficient mice showed increased CD24^−^CD73^+^ (Figure 5F) and CD44^+^CD45RB^+^ γδ thymocytes as well as an incremented number of γδ thymocytes producing IFN-γ and IL-4 cytokines (Figures S5C–S5E). The proportion of annexin V-positive γδ *Rag1Cre-E2*^*fl/fl*^ thymocytes was similarly reduced as upon total loss of NFATc1 compared to control cells (Figure S5F). The number of γδ thymocytes expressing CD4 co-receptor (8.5% *E2*^*fl/fl*^ and 42% *Rag1Cre-E2*^fl/fl^) (Figure 5G) and the Vγ1.1 and the Vδ6.3 gene segments increased significantly compared to control mice (Figure 5H). These results indicate that NKT γδ cells accumulate in the absence of the enhancer E2 and thereby the induction of NFATc1/α isoforms.

### NFATc1 binds to numerous genes expressed in γδ cells

**Figure 6:**
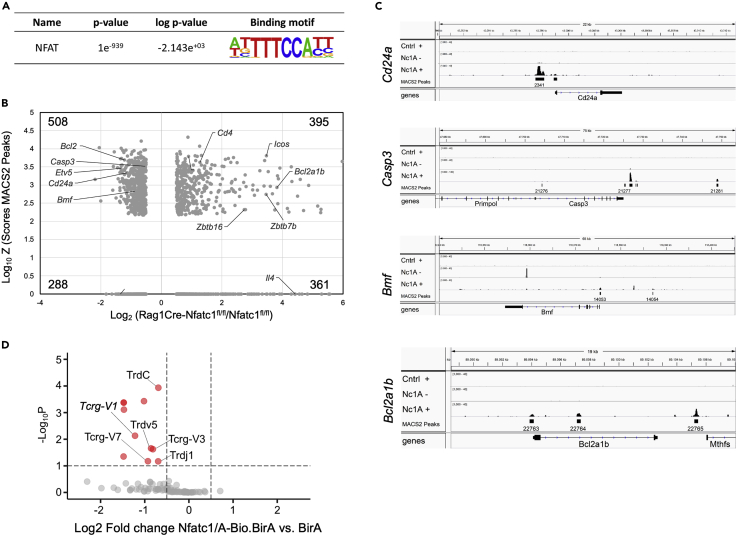
NFATc1 binds to genes expressed in γδ thymocytes (A) Motif enrichment in NFATc1-ChIP peaks identified in stimulated thymocytes from *Nfatc1/A-Bio.BirA* compared to *BirA* mice. (B) Vulcano plot of genes differentially expressed in isolated γδ thymocytes from *Nfatc1*^*fl/fl*^ and *Rag1Cre-Nfatc1*^*fl/fl*^ mice that contain NFATc1 binding sites at a distance of 100 kb around the transcriptional start site. Each dot is characterized by coordinates that represent the Log_10_ from the sum of scores that MACS2 assigned to each peak (on the y axis) and the Log_2_ of the fold change in expression of the indicated genes (on the x axis). The number of genes with or without peaks is indicated for each quadrant. (C) NFATc1 binding sites at and near the *Cd24a, Casp3, Bmf,* and *Bcl2a1b* loci. Annotated reads from ChIP-seq analysis for *BirA* (Cntrl) or *Nfatc1/A-Bio.BirA* (Nc1A) untreated (−) or stimulated with TPA and ionomycin for 4 h (+) are displayed together with the identified peaks by IGV 2.5.2. (D) Vulcano plot of differently expressed transcripts from freshly isolated *Nfatc1/A-Bio.BirA* and *BirA* DN thymocytes. Displayed are variable (V), joint (J), and constant (C) gene segments associated with TCRαβ and TCRγδ chains. Red dots mark significant differential expressed genes, and TCRγδ segments are highlighted with the name.

To elucidate whether NFATc1 binds prominent genes expressed in γδ thymocytes, we investigated the genome-wide binding sites of NFATc1 in thymocytes from transgenic *Nfatc1/A-Bio.BirA* mice in chromatin immunoprecipitation sequencing (ChIP-seq) assays. In those mice, an additional copy of NFATc1 is expressed from a bacterial artificial chromosome (BAC) transgene, bearing a biotin-tag at the C terminus of NFATc1/A^37^^,^^38^ of both isoforms NFATc1/α and NFATc1/β. The analysis of ChIP-seq results revealed the common NFAT binding motif (Figure 6A) within the peaks and allowed us to identify genes with NFATc1 binding sites enclosed by an area of 100 kb around their transcription start site. The Vulcano plot in Figure 6B shows that, compared to *Nfatc1*^*fl/fl*^, in γδ thymocytes from *Rag1Cre-Nfatc1*^*fl/fl*^ mice that are characterized by an expanded population of NKT γδ cells, 903 genes are differentially expressed and are a direct target of NFATc1 (indicated by log_10_ of MACS2 peaks score, Figure 6B), while 649 genes are not NFATc1 direct targets. Some examples of genes involved in the apoptosis/survival regulation of NKT γδ thymocytes are indicated, and the NFATc1 binding is shown in detail for the *Cd24a, Casp3, Bmf* (Bcl2-modifying factor)*,* and *Bcl2a1b* genes (Figure 6C). In addition, the RNA-seq data of thymocytes from *Nfatc1/A-Bio.BirA* mice expressing this extra copy of NFATc1 revealed that the expression of segments associated with the γδ TCRs, such as the *Tcrg*-V1 gene segment, was downregulated, in contrast to that of αβ TCR-associated gene segments (Figure 6D). These results indicate that overexpressing NFATc1 can suppress γδ transcripts.

## Discussion

In this study, we investigated the role of the transcription factor NFATc1 during the DN stages of thymocyte development. We show here that the absence of NFATc1 during these early stages of thymocytes affects the phenotypical and functional differentiation of γδ T cells. Ablation of NFATc1 in DN thymocytes leads to a marked increase in the number of NKT γδ cells that display high levels of the transcription factor PLZF, the “master regulator” of NKT cell development, and further characteristics of NKT cells.^15^

Using a *VavCre-Nfatc1*^*fl/fl*^ mouse model in which the depletion of NFATc1 occurs in hematopoietic progenitor cells, we observed a complete block of thymocyte development at the DN stages.^30^ However, in our study here, *Rag1Cre-Nfatc1*^*fl/fl*^ mice were generated for the specific ablation of NFATc1 starting at DN stages. They exhibited a very moderate decrease in overall thymic cellularity but a marked expansion of γδ thymocytes with NKT cell characteristics. Owing to the early and efficient activity of the Rag1Cre system in depleting NFATc1 function shortly ahead of γδ lineage choice, we suppose that this effect is cell intrinsic. This indicates that NFATc1 does not control the overall thymic T cell development but quite specific steps in the lineage determination of thymocytes.

In thymocytes, the signals delivered by the pre-TCR and TCR complexes differ from those in mature T cells. During development in the thymus, the pre-TCR (composed of a β and a pre-α chains) signals to the cell that the pre-T cell receptor components are properly expressed driving thymocyte survival, proliferation, and acquisition of a αβ T cell phenotype. This process is indicated as β-selection. Similarly, at this stage, some thymocytes might express a proper γδTCR licensing them to pass the γδ-selection process. Later in development in the thymus, the recognition of major histocompatibility complex molecules displaying a self-antigen by a mature TCR (composed of the β and α chains) signals whether thymocytes are selected positively or negatively. In peripheral mature T cells, in response to an antigen, TCR signaling leads predominantly to the activation of naive or resting T cells.

The current “signal strength theory” of thymocyte development claims that strong TCR signals, normally induced by a γδTCR, received by DN cells lead to the acquisition of a γδ lineage, whereas weaker signals, induced by the pre-TCR, support the generation of αβ thymocytes.^39^^,^^40^ Our data imply that strong signals that induce NFATc1 and its α-isoforms in parallel limit the number of PLZF^+^ CD4^+^ Vγ1.1^+^ Vδ6.3^+^ thymocyte populations. A similar increase in this population was described for *Itk*-, *Id3*-deficient, and SLP-76 mutant mice bearing thymocytes with impaired TCR signal strength.^16^^,^^35^^,^^41^

We hypothesized that the increase of NKT γδ population in *Rag1cre-Nfatc1*^*fl/fl*^ mouse reflects the acquisition of a better survival rate upon lack of NFATc1. Our RNA- and ChIP-seq analysis showed that genes involved in the apoptosis pathway, such as *Casp3* and *Bmf* (encoding the proapoptotic BH3-only Bcl-2 modifying factor),^42^ are downregulated in NFATc1-deficient γδ thymocytes. In addition, the surface marker CD24 is much less expressed on NFATc1-deficient γδ thymocytes, compared to control cells. It has been shown that the cross-link of CD24 with a specific antibody drives apoptosis of DN thymocytes.^43^ Therefore, in a physiological situation, the induction of those genes by NFATc1 will increase susceptibility to apoptosis. In line with this, our data revealed that the absence of NFATc1 leads to an upregulation of anti-apoptotic members of the Bcl2A1 family (*Bcl2a1a, Bcl2a1b, Bcl2a1d*). Bcl2A1 was described to enable the survival of DN thymocytes upon a proper pre-TCR signaling.^44^ However, a more detailed analysis of Bcl2A1 function by the complete ablation of all Bcl2A1 isoforms revealed no obvious defects in thymocyte development but a decrease in γδ T cell numbers in the spleen.^45^ On the other hand, a positive correlation between the expression of NFATc1 and Bcl2A1-mediated cell survival has been observed in peripheral lymphocytes,^26^^,^^46^ suggesting the existence of an alternate mechanism for the survival of thymocytes and peripheral T (and B) cells.

Among the different effector types of γδ cells, NKT γδ cells share several characteristics with NKT αβ cells, such as their surface markers, transcription factors, and cytokine production. PLZF is the “master regulator” of several innate-like T cells, including NKT cells.^18^ Previous data have shown that PLZF in NKT cells is induced by a strong TCR signals.^17^ In our experiments, dampening the intracellular signal transfer from the TCR by inactivation of NFATc1 in the DN thymocytes leads to an increased number of PLZF-expressing γδ thymocytes. Reinforcing TCR signaling by stimulation with αCD3 provokes a decrease in PLZF^+^ *Nfatc1*^*−/−*^ deficient γδ thymocytes. This decrease could be abolished by CsA, an inhibitor of the Ca^2+^/calcineurin network that controls the activation of NFAT factors. These findings suggest that Ca^2+^/calcineurin/NFAT signals limit the number of NKT γδ thymocytes and, thereby, restrict the development of those cells. Future experiments using transgenic γδ TCR models aim to underpin this conclusion.^39^^,^^47^

The transcription factor ThPOK (coded by the *Zbtb7b* gene) is required to drive immature thymocytes toward the CD4 lineage,^48^^,^^49^ and it has been shown that PLZF-expressing Vγ1.1^+^Vδ6.3^+^ T cells also express the CD4 co-receptor.^16^ These observations were confirmed in our analysis, which revealed a substantial increase in CD4^+^ Vγ1.1^+^Vδ6.3^+^ γδ thymocytes. Noteworthy, in our RNA-seq analysis, a marked upregulation of the *Zbtb7b* gene in *Nfatc1*^*−/−*^ γδ thymocytes was observed. In addition, our ChIP-seq assays showed the binding of NFATc1 at the *Zbtb7b* locus, indicating a direct transcriptional regulation by NFATc1.

The observation that γδ thymocytes express higher levels of NFATc1 compared to αβ thymocytes led us to assume that, as in peripheral T cells, the induction of NFATc1/α isoforms contributes to the high NFATc1 expression level.^28^ Indeed, in the thymus of *Rag1Cre-E2*^*fl/fl*^ mice, in which the deleted enhancer E2 leads to the loss of α isoforms, a similar accumulation of NKT γδ was detected. This suggests that γδ thymocytes require strong signals for the tight control of NKT γδ cell numbers. Taken together, the constitutive expression of NFATc1 in DN thymocytes plays a minor role in their further development, but the induction of NFATc1/α isoforms, regulated by the distal enhancer E2, controls the proper development of NKT γδ cells.

### Limitations of the study

We have to acknowledge some limitations of our study. We are aware of the importance of further signaling pathways for γδ thymocyte development such as the one mediated by the signaling lymphocytic activation molecule (SLAM) receptors. Because the differentiation of thymocytes requires the integration of several signaling pathways together with TCR signaling, this has to be addressed using additional genetic models in the future.

## STAR★Methods

### Key resources table

**Table:** 

REAGENT or RESOURCE	SOURCE	IDENTIFIER
**Antibodies**		

Rat monoclonal anti-mouse CD4	BioLegend	Cat# 100427; RRID:AB_493646
Rat monoclonal anti-mouse CD8a	BioLegend	Cat# 100752; RRID:AB_2563057
Rat monoclonal anti-mouse CD25	BioLegend	Cat# 102007; RRID:AB_312856
Rat monoclonal anti-mouse/human CD44	BioLegend	Cat# 103005; RRID:AB_312956
Armenian Hamster monoclonal anti-mouse TCR beta chain	BioLegend	Cat# 109207; RRID:AB_313430
Armenian Hamster monoclonal anti-mouse TCR gamma/delta	BioLegend	Cat# 118105; RRID:AB_313829
Rat monoclonal anti-mouse CD24	BioLegend	Cat# 101805; RRID:AB_312838
Rat monoclonal anti-mouse CD73	BioLegend	Cat# 127205; RRID:AB_1089065
Rat monoclonal anti-mouse CD44	BioLegend	Cat# 103011; RRID:AB_312962
Rat monoclonal anti-mouse CD45RB	BioLegend	Cat# 103313; RRID:AB_1953290
Mouse monoclonal anti-mouse CD45.1	BioLegend	Cat# 110730; RRID:AB_1134168
Mouse monoclonal anti-mouse CD45.2	BioLegend	Cat# 109814; RRID:AB_389211
Rat monoclonal anti-mouse IFN-gamma	BioLegend	Cat# 505821; RRID:AB_961361
Rat monoclonal anti-mouse IL-4	BioLegend	Cat# 504105; RRID:AB_315319
Rat monoclonal anti-mouse IL-17A	BioLegend	Cat# 506903; RRID:AB_315463
Mouse monoclonal anti-mouse NK-1.1	BioLegend	Cat# 108723; RRID:AB_830870
Armenian Hamster monoclonal anti-mouse TCR Vgamma1.1 Vgamma1.2	BioLegend	Cat# 142703; RRID:AB_10960739
Rat monoclonal anti-mouse TCR Vdelta6.3	BioLegend	Cat# 154805; RRID:AB_2728220
Armenian Hamster monoclonal anti-mouse PLZF	BioLegend	Cat# 145803; RRID:AB_2561966
Armenian Hamster monoclonal anti-mouse CD3epsilon	BioLegend	Cat# 100301; RRID:AB_312666
Mouse monoclonal anti Bcl2	BioLegend	Cat# 658707; RRID:AB_2563281
Mouse monoclonal anti Ki-67	BioLegend	Cat# 652405; RRID:AB_2561929
Mouse monoclonal anti Bcl-xL	Santa Cruz Biotechnology	Cat# sc-8392; RRID:AB_626739
Mouse monoclonal anti NF-ATc1	BD Pharmingen	Cat# 556602; RRID:AB_396478
Mouse monoclonal beta Actin Loading Control	Thermo Fisher Scientific	Cat# MA5-15739; RRID:AB_10979409

**Chemicals, peptides, and recombinant proteins**		

Cyclosporin A	Sigma-Aldrich	Cat# 239835; CAS 59865-13-3
Phorbol 12-myristate 13-acetate	Sigma-Aldrich	Cat# P1585; CAS 16561-29-8
Ionomycin	Thermo Fisher Scientific	Cat# I24222; CAS 56092-82-1
APC Annexin V	Biolegend	Cat# 640919
Mouse CD1d PBS-57 tetramer, BV421	NIH Tetramer Core Facility	N/A
mCD1d control	NIH Tetramer Core Facility	N/A

**Critical commercial assays**		

eBioscience™ Foxp3/Transcription Factor Staining Buffer Set	Thermo Fisher Scientific	Cat# 00-5523-00
eBioscience Intracellular Fixation & Permeabilization Buffer Set	Thermo Fisher Scientific	Cat# 88-8824-00
ChIP DNA Clean & Concentrator	Zymo Research	Cat# D5205
NEBNext® Ultra™ II DNA Library Prep kit for Illumina	NEB	Cat# E7645S
HiSeq Rapid SBS Kit v2	Illumina	Cat# FC-402-4022
RNeasy Plus Micro Kit	Qiagen	Cat# 74034
NEBNext® Poly(A) mRNA Magnetic Isolation Module	NEB	Cat# E7490S
NEBNext® Ultra™ II RNA Library Prep Kit for Illumina	NEB	Cat# E7770S
TruSeq Rapid Cluster Kit	Illumina	Cat# GD-402-400

**Deposited data**		

Raw and analyzed data	This paper	GSE198031

**Experimental models: Organisms/strains**		

Mouse: B6.Cg-Nfatc1tm3Glm/Aoa/J	Jackson Laboratory	RRID:IMSR_JAX:022786
Mouse: B6;-Rag1tm1(cre)Thr/J	McCormack et al.^50^	MGI:3584018
Mouse: B6.Tg(Nfatc1-EGFP)#Srf/J	Bhattacharyya et al.^36^	MGI:5295241
Mouse: B6.Tg(Nfatc1)1Srf/J	Klein-Hessling et al.^37^	MGI:7335972
Mouse: B6.Gt(ROSA)26Sortm1(birA)Mejr	Driegen et al.^38^	RRID:IMSR_JAX:010920
Mouse: B6.SJL-Ptprca Pepcb/Boy/J	Jackson Laboratory	RRID:IMSR_JAX:002014
Mouse: C;129S4-Rag2tm1.1Flv Il2rgtm1.1Flv/J	Jackson Laboratory	RRID:IMSR_JAX:01459
Mouse: B6.Cg-Nfatc1em1Serf /J	This paper	MGI:7428886

**Oligonucleotides**		

*Actb* fw TGTCCACCTTCCAGCAGATGT	This paper	N/A
*Actb* rev AGCTCAGTAACAGTCCGCCTAG	This paper	N/A
*B2m* fw CTGCTACGTAACACAGTTCCACCC	This paper	N/A
*B2m* rev CATGATGCTTGATCACATGTCTCG	This paper	N/A
*Nfatc1* E1-E3 fw GGGAGCGGAGAAACTTTGC	This paper	N/A
*Nfatc1* E1-E3 rev CAGGGTCGAGGTGACACTAGG	This paper	N/A
*Nfatc1* E2-E3 fw AGGACCCGGAGTTCGACTTC	This paper	N/A
*Nfatc1* E2-E3 rev GCAGGGTCGAGGTGACACTAGG	This paper	N/A
*Nfatc2* fw GGGTTCGGTGAGTGACAGTT	This paper	N/A
*Nfatc2* rev CTCCTTGGCTGTTTGGGATA	This paper	N/A
*Nfatc3* fw CCGATGACTACTGCAAACTGTGG	This paper	N/A
*Nfatc3* rev TTTGAATACTTGGGCACTCAAAGG	This paper	N/A
*Bcl2* fw GGCGCCCCTGGGGGCTGCCC	This paper	N/A
*Bcl2* rev ACCTGCAGTTCAAAACACCTCT	This paper	N/A
*Bcl2a1a* fw GATACGGCAGAATGGAGGTT	This paper	N/A
*Bcl2a1a* rev GAAAGAGCATTTCCCAGATC	This paper	N/A
*Bcl2l1* fw ACAAGGAGATGCAGGTATTGG	This paper	N/A
*Bcl2l1* rev CCACAAAAGTGTCCCAGCCGC	This paper	N/A
*Zbtb16* fw CCCAGTTCTCAAAGGAGGATG	This paper	N/A
*Zbtb16* rev TTCCCACACAGCAGACAGAAG	This paper	N/A

**Software and algorithms**		

FlowJo 10.8.1	BD Biosciences	https://www.flowjo.com/solutions/flowjo/downloads
Map with Bowtie for Illumina (version 1.1.2)	Langmead et al.^51^	http://bowtie-bio.sourceforge.net/index.shtml
MACS2 callpeak	Feng et al.^52^	https://github.com/macs3-project/MACS
GREAT (version 4.0.4)	McLean et al.^53^	http://bejerano.stanford.edu/great/public/html/index.php
Integrative Genomics Viewer IGV (version 2.5.2)	Robinson et al.^54^	https://software.broadinstitute.org/software/igv/home
STAR (v.2.7)	Dobin et al.^55^	https://bioinformaticshome.com/tools/rna-seq/descriptions/STAR.html
Rsubread: Mapping, quantification and variant analysis of sequencing data	Liao et al.^56^	https://rdrr.io/bioc/Rsubread/
DESeq2	Love et al.^57^	http://www.bioconductor.org/packages/release/bioc/html/DESeq2.html
pHeatmap	Kolde R., 2019	https://cran.r-project.org/package=pheatmap
EnhancedVolcano (version 1.12.0)	Bioconductor	https://bioconductor.org/packages/devel/bioc/vignettes/EnhancedVolcano/inst/doc/EnhancedVolcano.html
Enrichr tool	Chen et al.^58^	https://maayanlab.cloud/Enrichr/
Metascape, Gene Annotation & Analysis Resource	Zhou et al.^59^	http://metascape.org/gp/index.html#/main/step1
Prism	GraphPad	https://www.graphpad.com/scientific-software/prism/

### Resource availability

#### Lead contact

Further information and reasonable requests for resources and reagents should be directed to the lead contact, Stefan Klein-Hessling (stefan.klein-hessling@uni-wuerzburg.de).

#### Materials availability

The mouse line C57BL/6J Nfatc1 E2^fl/fl^ generated in this study has been deposited to the Knockout Mouse Project (KOMP), MGI:7428886 (synonym Nfatc1^em1Serf^).

### Experimental model and subject details

#### Mice and treatment

All mice used in the experiments were 4-6 weeks old, at C57/B6 background and sex- and age-matched. The B6.*Nfatc1-eGfp-Bac* and the B6.*Nfatc1/A-Bio.BirA* mice have been previously described.^29^^,^^36^^,^^37^^,^^38^ *Rag1Cre-Nfatc1*^*fl/fl*^ mice were generated by breeding mice bearing *Nfatc1* alleles with loxP-flanked exon 3 *(Nfatc1*^*fl/fl*^ mice)^60^ with mice that express Cre recombinase under the control of the *Rag1* locus.^50^ The C57BL/6J *Nfatc1 E2*^*fl/fl*^ mice contain two loxP sites flanking the 1285 bp enhancer E2 (mm9: chr18:80808265-80809549) in intron 10 of the *Nfatc1* gene. CRISPR/Cas9 mediated generation of the *Nfatc1 E2*^*fl/fl*^ mice (MAGEC laboratory, WEHI, Melbourne, Australia) was performed as previously described.^61^ Briefly, *Cas9* mRNA (20 ng/μl) and sgRNAs (10 ng/μl of the sequences CCAGATCTTTGGTCACTTGT and CTGACTTGCAGTGTCTAGTT) were injected together with a targeting vector (Figure S5) for homologous recombination into the cytoplasm of fertilized one-cell stage C57BL6/J embryos. After 24 h, two-cell stage embryos were transferred into the uteri of pseudo-pregnant female C57BL/6J mice. DNA from viable pups was screened in two PCR reactions. The primers for the *Nfatc1* lox1 PCR are CTTCAGAGGCCGAGCTAGAG (forward) and TTTCAGGTGCCCAAGAGAGC (reverse). The primers for the *Nfatc1* lox2 PCR are GTAGGCAGCTCTGGGGAATG (forward) and CCCACTGTTCTCATCCCACC (reverse). To generate bone marrow chimeric mice sub-lethally (6 Gy) irradiated Rag2^-/–^ γc^–/–^ host mice (JAX stock #014593,^62^)were injected retro-orbitally with 1x10^7^ CD45.1^+^CD45.2^+^ WT (JAX stock #002014,^63^ together with 1x10^7^ CD45.2^+^ *Rag1Cre-Nfatc1*^*fl/fl*^ bone marrow cells. Analyses of thymocytes was performed six weeks after reconstitution. All mice were maintained in the central animal facility (ZEMM) of the University of Würzburg, according to the institutional guidelines.

### Method details

#### Cell isolation

Thymi, axillary, inguinal, and brachial lymph nodes, and spleen from 4- to 6-week-old mice were homogenized by mechanical disaggregation in PBS containing 0.1% BSA and were passaged through a 70 μm filter (Falcon^TM^, Fisher Scientific GmbH, D-58239 Schwerte). Splenocyte suspensions were incubated with red blood cell lysis buffer (©2022 Merck KGaA, Darmstadt, DE).

DN thymocyte selection was carried out using MACS magnetic separation with anti-CD4 (L3T4) and anti-CD8 (Ly-2) microbeads from Miltenyi Biotec according to the manufacturer’s protocol. γδ thymocytes were isolated using the TCRγ/δ^+^ T cell isolation kit, from Miltenyi Biotec according to the manufacturer’s protocol.

Single-cell suspensions from ear skin were prepared using Accumax (Merck KGaA) according to the manufacturer’s protocol. The tissue was dissociated using C-tubes and gentleMACS Octo Dissociator (Miltenyi Biotec). Single-cell suspensions were prepared after the passage of the dissociated tissue through a 70 μm cell strainer and rinsed by RPMI (Gibco^TM^, Thermo Fisher Scientific, MA USA) with 10 % FBS (Gibco^TM^, Thermo Fisher Scientific, MA, USA).

For isolation of intestinal lymphocytes, the small intestine and colon were excised, flushed, and opened longitudinally. Intestines were cut into pieces of 0.5 cm in length and were incubated in HBSS (Thermo Fisher Scientific) with EDTA and DTT for isolation of intraepithelial lymphocytes (IELs). Intestinal tissue was enzymatically digested with collagenase D and DNase I (Merck KGaA) for the isolation of lamina propria lymphocytes. Isolated cells were resuspended in FACS buffer for staining.

#### RNA isolation, real-time PCR analysis, and immunoblotting

RNA was extracted from freshly isolated thymocytes*.* Depending on the number of cells, they were lysed in 350 μl or 600 μl of RLT buffer supplemented with β-mercaptoethanol (10 μl/ml) of RNeasy Plus Mini kit (QIAGEN). Further steps proceeded according to the manufacturer’s protocol. RNA concentrations were measured using a Nanodrop spectrophotometer (Peqlab), and an RNA amount between 100 ng and 2.5 μg was used to generate cDNAs.

Complementary DNA (cDNA) was generated using the Superscript^TM^ IV VILO kit (Thermo Fisher Scientific). The reverse-transcribed RNA was used for quantitative PCR analysis using the SYBR green master mix (Applied Biosystem) under the following conditions: pre-incubation 95 °C for 5 min, amplification 95 °C for 10 sec, 60 °C for 30 sec, 72 °C for 30 sec, melting curve 95 °C for 5 sec, 65 °C for 1 min, 97 °C continuous, cooling 40 °C for 10 sec.

Gene expression was normalized to the expression of the control genes *Actb* (encoding β-actin) or *B2m* (b2-microtubulin) as indicated. The relative expression was calculated by the change-in-cycling threshold (ΔΔCt) method. Primer sequences are listed in key resources table.

Immune blots were performed from whole protein extracts prepared with RIPA buffer on PAGE-SDS gels followed by detection of NFATc1 using the 7A6 mAb #556602 (BD Pharmingen). As a loading control, filters were re-probed with the mAb # A2066 (Sigma-Aldrich) specific for β-actin. Signals were developed using goat anti-mouse IgG-HRP conjugate (Invitrogen) and a chemiluminescence detection system (Thermo Fisher Scientific).

#### Stimulation of thymocytes

Total thymocytes from *Rag1Cre-Nfatc1*^*fl/fl*^ and *Nfatc1*^*fl/fl*^ mice were left unstimulated or cultivated in complete RPMI containing 10 % FCS on αCD3 plate-bound 48 wells, at 37 °C with 5 % CO_2_ saturation for 24 h, without or with the addition of 100 ng/ml cyclosporin A (CsA). Alternative stimulation was done with 100 ng/ml 12-O-Tetradecanoylphorbol-13-acetate (TPA) and 0,5 μM ionomycin when indicated.

#### Antibodies and flow cytometry

All antibodies used in this study were from Biolegend and anti-mouse: purified anti-CD16/anti-CD32, CD4 (GK1.5), CD8 (53-66.7), CD25 (PC61), CD44 (IM7), TCRβ (H57-597), TCRδ (GL3), CD24 (M1/69), CD73 (TY/11.8), CD45RB (C363-16A), IFN-γ (XMG1.2), IL-4 (11B11), IL-17 (TC11-18H10.1), PLZF (9E12), NK1.1 (PK136), Vγ1.1 (2.11), Vδ6.3 (C504.17C), CD45.1 (A20), CD45.2 (104), ki67 (16A8), Zombie Aqua™. Cell death analysis was performed by annexin V staining. The PBS57-loaded mouse BV421-conjugated CD1d tetramer and unloaded control were obtained from the NIH Tetramer Core Facility, USA. Flow cytometry assays were performed according to standard protocols. Intracellular staining for transcription factors was carried out using the Foxp3 transcription factor staining buffer set (eBioscience^TM^, San Diego, CA). Intracellular staining for IL-4, IFN-γ, and IL-17 was performed using the Intracellular Fixation & Permeabilization Buffer Set (eBioscience^TM^). Cytokine production was induced by incubating cells with 0,5μM ionomycin (Thermo Fisher) and 100 ng/ml of phorbol 12-myristate 13-acetate (PMA) (Sigma-Aldrich) incubated at 37 °C with 5 % CO_2_ saturation for 5 h, with brefeldin A solution 1000X (Biolegend, San Diego, CA). The cells were acquired using a FACS Canto flow cytometer and analyzed with FlowJo^TM^ Software. Whenever the y-axis is labeled with “count”, the cells were analyzed by SSC-H.

#### ChIP-seq analysis

For purification affinity-based chromatin immunoprecipitation and sequencing (ChIP-seq) experiments, 3-5 x10^6^/ml total thymocytes from control *BirA* and *Nfatc1/A-Bio.BirA* mice were cultured in complete RPMI (Gibco^TM^, Thermo Fisher Scientific) containing 10 % FBS (Gibco^TM^, Thermo Fisher Scientific) in the presence of TPA (100 ng/ml) and ionomycin (0,5 μM) for 4 h or left untreated. Chromatin from 10^7^ thymocytes was obtained upon fixation with 1 % formaldehyde, followed by lysis in 1 % SDS-containing buffer with the addition of the serine protease inhibitor, PMSF (1 mM). Chromosomal DNA fragments in length between 200-800 bp were obtained by sonication for 10 min (35 % amplitude, 30 sec of pulse, and 30 sec of pause), with a Vibra-Cell VCX 130 sonicator (Sonics, CT, USA). As described, chromatin fragments were precipitated using streptavidin-coupled Dynabeads M-280 (M-280, Thermo Fisher Scientific, MA, USA).^37^

DNA fragments were purified using the ChIP DNA Clean & Concentrator kit (ZYMO Research), and 3 ng of precipitated DNA from *BirA* and *Nfatc1/A-Bio.BirA* samples were used for library preparation using NEBNext® Ultra™ II DNA Library Prep kit for Illumina® according to the manufacturer’s protocol. The quantity of library was assessed by Qubit 2.0 using a dsDNA HS assay kit (Thermo Fisher Scientific, USA, Q32851), and the quality was determined on an Agilent’s Bioanalyzer 2100 using a high sensitivity DNA chip (Agilent Technologies, CA, USA).

Sequencing of the final libraries was carried out as a 59 bp single read run on an Illumina HiSeqTM2500 (Illumina, San Diego, USA) by the Institute of Molecular Genetics (JGU, Mainz, Germany) using a TruSeq Rapid SBS Kit v2 and a HiSeq Rapid Flow Cell v2. 59 bp sequence reads that passed the Illumina quality filtering were aligned to the mouse genome assembly version of July 2007 (NCB I37/mm9) using the Map with Bowtie for Illumina (version 1.1.2).^51^ Peak calling was done using MACS on Galaxy (MACS version 2.1.1.)^52^ with default parameters, a genome size of 2,700,000,000 bp (mm9), and the reads from Rosa26BirA Cntrl^+^ IP as a control sample. Genes nearby 100 kb to annotated peaks were identified with GREAT version 4.0.4.^53^ Results were visualized with the “Integrative Genomics Viewer” IGV version 2.5.2.^54^

#### Preparation of cDNA libraries and NGS RNA sequencing

According to the manufacturer's protocol, the RNA was purified with the RNeasy Plus Micro kit (QIAGEN). The RNA was quantified with a Qubit 2.0 fluorometer from Invitrogen and the quality was assessed on a Bioanalyzer 2100 using an RNA 6000 Pico chip, both from Agilent. Only samples with an RNA integrity number (RIN) of > 8 were used for the cDNA library preparation. Barcoded mRNA-seq cDNA libraries were prepared from 50 ng of total RNA using the NEBNext® Poly(A) mRNA Magnetic Isolation Module and NEBNext® Ultra™ II RNA Library Prep kit for Illumina® according to the manual. The quantity was assessed using Invitrogen’s Qubit HS assay Kit, and library size was determined using Agilent’s 2100 Bioanalyzer HS DNA assay.

Barcoded RNA-Seq libraries were clustered using HiSeq® Rapid SR Cluster Kit v2 using 8 pM and 59 bps were sequenced on the Illumina HiSeq2500 using HiSeq® Rapid SBS Kit v2 (59 cycles). The quality of the obtained FASTQ files was controlled with FastQC, the samples with corresponding controls were trimmed for the adaptor and first 10 nucleotide sequences with cutadapt,^64^ and all files were aligned using STAR (v.2.7) to the GRCm38.98 reference genome using standard settings.^55^ The aligned data were counted in R using the featureCounts function of the Rsubread package.^56^ DESeq2 was used for the differential gene expression analysis.^57^ Significant differentially expressed genes were defined as having an adjusted p-value < 0.01 and log_2_ Fold Change > 0.5 and log_2_ Fold Change < −0.5. Significant differentially expressed genes were visualized using the pHeatmap package in R (https://CRAN.R-project.org/package=pheatmap) for the *Rag1Cre-Nfatc1*^*fl/fl*^ and control samples. All expressed TCR-segment genes were identified in the *Nfatc1/A-Bio.BirA* samples were displayed using EnhancedVolcano (R package version 1.12.0, https://github.com/kevinblighe/EnhancedVolcano). For unsupervised cell identity analysis, the significant differentially expressed genes of *Rag1Cre-Nfatc1*^*fl/fl*^ samples and controls were analyzed using the Enrichr tool.^58^^,^^65^ The PanglaoDB Augmented 2021 database was used as a reference to test for enrichment with standard Enrichr settings. The P-value was calculated from Fisher’s exact test. Pathway and process enrichment analysis has been carried out using the Metascape Tool^59^ with a p-value < 0.01, a minimum count of 3, and an enrichment factor > 1.5.

### Quantification and statistical analysis

Statistical analyses were performed using GraphPad 5.0 (Prism) software. All the results are indicated as the mean ± SEM. A confidence level of 95 % level was used, and the statistical significance was determined by unpaired student’s t-tests. Significant differences between data were indicated with ∗p-value < 0.05, ∗∗p-value < 0.005, ∗∗∗p-value < 0.001, ∗∗∗∗p-value <0.0001.

## Data Availability

ChIP-Seq and RNA-Seq data supporting this study are available in the NCBI’s Gene Expression Omnibus and are accessible through the GEO series accession number GSE198031. Any additional information required to reanalyze the data reported in this paper is available from the lead contact upon request. This paper does not report the original code.
