# Supplementary material for: NFATc1 induction by an intronic enhancer restricts NKT γδ cell formation
Source: iScience. 2023 Feb 19;26(3):106234. doi: 10.1016/j.isci.2023.106234 (PMC10011748; doi:10.1016/j.isci.2023.106234)
Supplement: Document S1. Figures S1–S6 [file mmc1.pdf]

iScience, Volume *26*

## Supplemental information

# NFATc1 induction by an intronic enhancer restricts NKT $\gamma\delta$ cell formation

**Sabrina Giampaolo, Cristina M. Chiarolla, Konrad Knöpper, Martin Vaeth, Matthias Klein, Azeem Muhammad, Tobias Bopp, Friederike Berberich-Siebelt, Amiya K. Patra, Edgar Serfling, and Stefan Klein-Hessling**

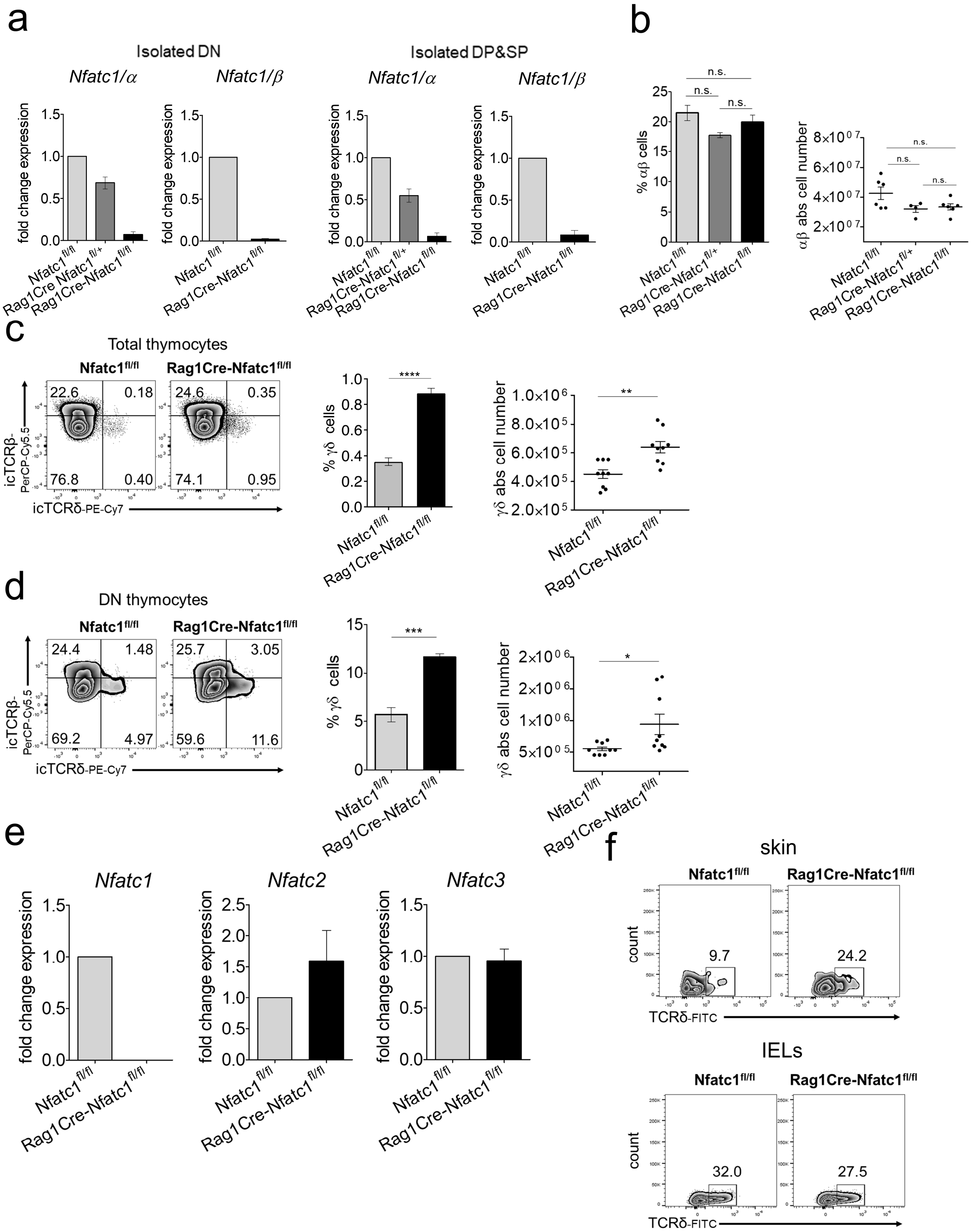


**Supplementary Figure 1. NFATc1 ablation and increase of γδ cells in the DN thymocyte compartment. Related to Figure 1**

**a**) Left, qRT-PCR assays of *Nfatc1* expression in DN thymocytes from *Nfatc1$^{fl/fl}$*, *Rag1Cre-Nfatc1$^{fl/+}$*, and *Rag1Cre-Nfatc1$^{fl/fl}$* mice. The expression of both the α and β isoforms of *Nfatc1* was checked using specific primers pairs, exons E1-E3 for (the α forms), and E2-E3 exons (for β forms). Right, qRT-PCR of *Nfatc1* expression in freshly isolated DP and SP subsets from *Nfatc1$^{fl/fl}$*, *Rag1Cre-Nfatc1$^{fl/+}$*, and *Rag1Cre-Nfatc1$^{fl/fl}$* thymocytes. Expression levels were normalized relative to the housekeeping *Actb* gene and are shown as fold change relative to control. **b**) Flow cytometry analysis of thymocytes from *Nfatc1$^{fl/fl}$* , *Rag1Cre-Nfatc1$^{fl/+}$* and *Rag1Cre-Nfatc1$^{fl/fl}$* mice. Total thymocytes gated on living cells were stained with anti-TCRδ and anti-TCRβ as in Figure 1d. Percentages (left) and absolute numbers (right) of αβ T cells in the thymi of mice are shown. Each dot represents one mouse. **c**) Total thymocytes from

*Nfatc1^{fl/fl}* and *Rag1Cre-Nfatc1^{fl/fl}* mice were stained intracellularly with anti-TCRδ and anti-TCRβ. Percentages (mid) and absolute numbers (right) of TCRδ⁺ thymocytes in mice are indicated. **d**) Thymocytes stained as in (c) were gated for the DN cells. Percentages (mid) and absolute numbers (right) of TCRδ⁺ thymocytes in the DN population in *Nfatc1^{fl/fl}* and *Rag1Cre-Nfatc1^{fl/fl}* mice are indicated. **e**) RT-PCR analysis of *Nfatc1, Nfatc2,* and *Nfatc3* transcripts in isolated γδ thymocytes from *Rag1Cre-Nfatc1^{fl/fl}* and control mice. Expression levels were normalized relative to the housekeeper *Actb* gene and are shown as fold change relative to control. **f**) Representative flow cytometry plot of the accumulation of γδ T cells in the skin (above) and IELs (below) from *Nfatc1^{fl/fl}* and *Rag1Cre-Nfatc1^{fl/fl}* mice. Data from at least five independent experiments with three or four mice for each genotype are shown as mean ± SEM. The statistical significance was determined by unpaired student's t-tests. *p-value < 0.05, **p-value < 0.005, ***p-value < 0.001, n.s not significant.

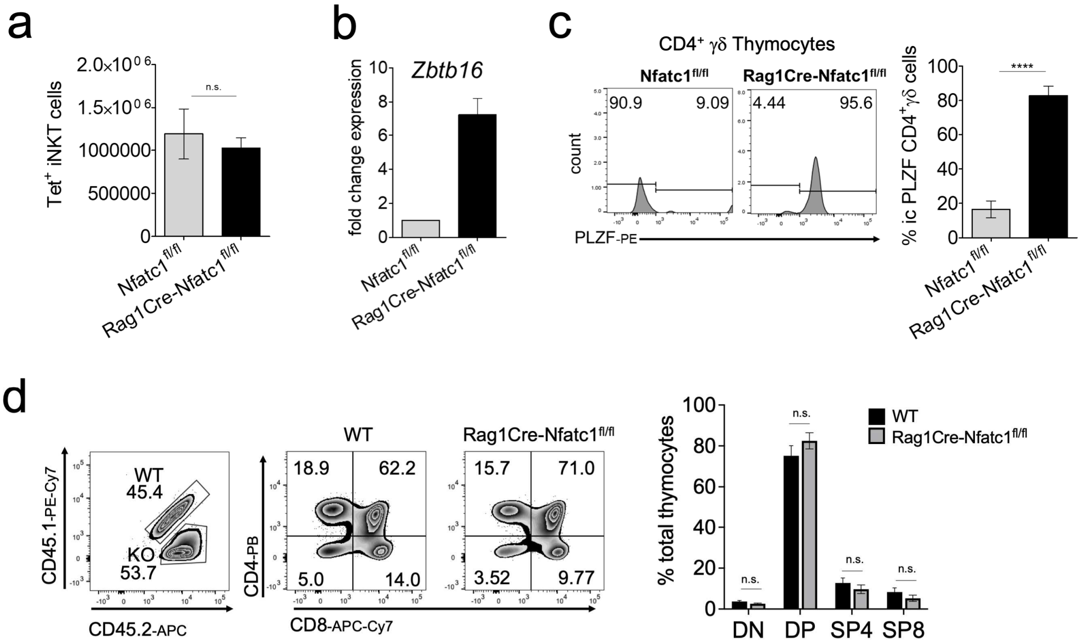


**Supplementary Figure 2. Expression of PLZF transcription factor in WT and NFATc1-deficient γδ thymocytes. Related to Figure 3**

**a**) Absolute cell numbers of tetramer-positive (Tet⁺) NKT αβ cells in isolated thymocytes from *Nfatc1^{fl/fl} and Rag1Cre-Nfatc1^{fl/fl}* mice. **b**) qRT-PCR assay showing the fold change of *Zbtb16* (encoding PLZF) expression in freshly isolated DN from *Rag1Cre-Nfatc1^{fl/fl}* compared to *Nfatc1^{fl/fl}* control mice. **c**) (Left) gated CD4⁺ γδ thymocytes from *Rag1Cre-Nfatc1^{fl/fl}* and *Nfatc1^{fl/fl}* mice positively stained with anti-PLZF. (Right) percentages of PLZF⁺ CD4⁺ γδ thymocytes. **d**) (Left) flow cytometry analysis of thymocytes from WT and *Rag1cre-Nfatc1^{fl/fl}* bone marrow chimera. The WT and *Rag1Cre-Nfatc1^{fl/fl}* thymic compartments were analyzed for CD4 and CD8 expression. (Right) Comparison of percentages of the four thymic populations from the WT and the *Rag1Cre-Nfatc1^{fl/fl}* compartments in bone marrow chimera. Data from at least three independent experiments are shown as mean ± SEM. The statistical significance was determined by unpaired student's t-tests. ****p-value <0.0001, n.s. not significant.

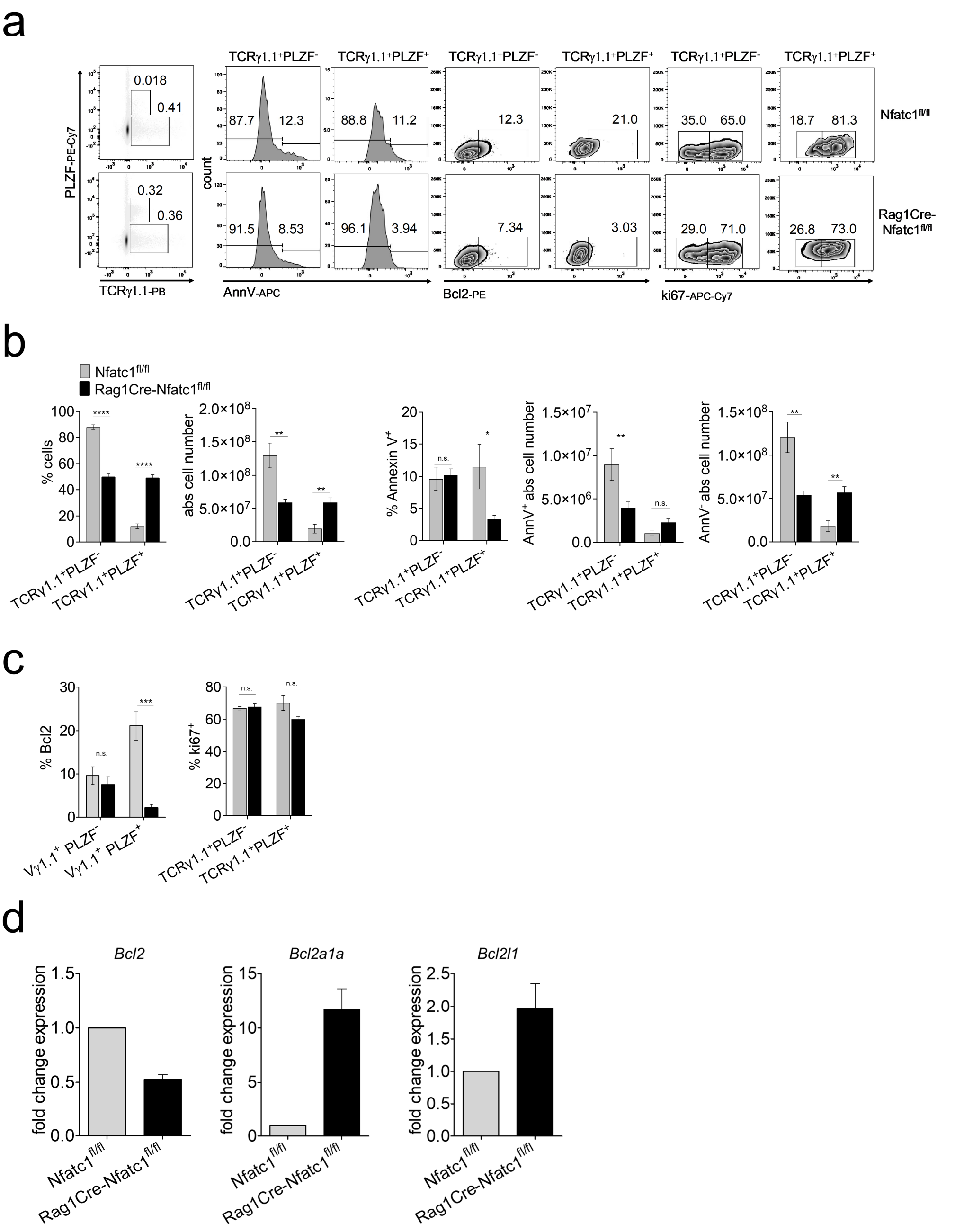


**Supplementary Figure 3. The absence of NFATc1 reduces cell death and Bcl-2 but not Ki-67 expression in TCRγ1.1+ PLZF+ thymocytes. Related to Figure 4**

**a**) Flow cytometry analysis of *Nfatc1^fl/fl^* and *RagCreNfatc1^fl/fl^* thymocytes stained with anti-TCRγ1.1 and anti-PLZF, annexin V, Bcl2, and ki67. The population of TCRγ1.1$^+$ cells was gated for PLZF$^-$ and PLZF$^+$ populations and analyzed for the expression of those factors. **b**) (Left) percentages and absolute cell numbers of TCRγ1.1$^+$ PLZF$^-$ and PLZF$^+$ populations, and (right) percentages of annexin V positive and absolute cell numbers of annexin$^-$ and annexin$^+$ TCRγ1.1$^+$ PLZF$^-$ and PLZF$^+$ populations calculated according to the analysis in a). **c**) Percentages of Bcl-2$^+$ and Ki-67$^+$ in TCRγ1.1$^+$ PLZF$^-$ and PLZF$^+$ populations. **d**) Changes of *Bcl2, Bcl2a1a,* and *Bcl2l1* transcripts in freshly isolated γδ thymocytes from *Nfatc1$^{fl/fl}$ and Rag1Cre-Nfatc1$^{fl/fl}$* mice analyzed by qRT-PCR. Gene expression was normalized relative to the *Actb* gene and calculated as fold change, relative to the gene expression in thymocytes from *Nfatc1$^{fl/fl}$* control mice. Data from at least two independent experiments are shown as mean ± SEM. The statistical significance was determined by unpaired student's t-tests. *p-value < 0.05, ***p-value < 0.001, n.s. not significant.

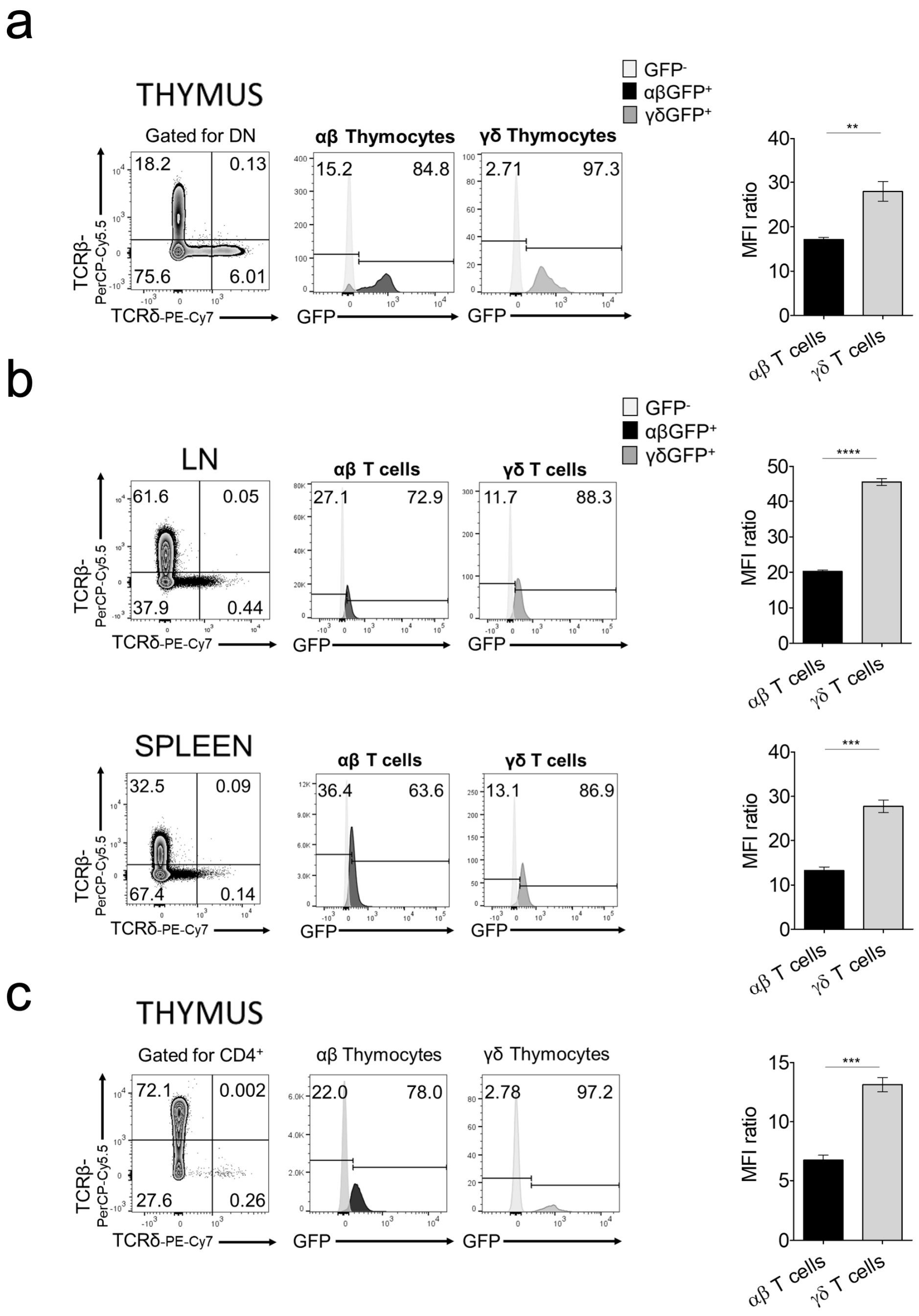


**Supplementary Figure 4. Strong NFATc1 expression in NKT γδ cells. Related to Figure 5**
**a**) Thymocytes from *Nfatc1-Egfp* mice (stained as in Figure 5a) were gated for DN cells, and the percentages of GFP-expressing αβ and γδ thymocytes were calculated in the DN compartment. **b**) Above, the percentage of cells expressing GFP in lymph nodes from *Nfatc1-Egfp* reporter mice is reported. Lymphocytes were stained with anti-TCRβ and anti-TCRδ. Left, the percentage of GFP positive αβ vs γδ lymphocytes from *Nfatc1-EGfp* reporter mice. Right, MFI ratio in αβ and γδ lymphocytes. Below, cells were stained with anti-TCRβ and anti-TCRδ , and the expression of GFP in splenocytes from *Nfatc1-Egfp* mice was measured. Left, the percentage of GFP positive αβ vs γδ splenocytes from *Nfatc1-EGfp* mice. Right, GFP MFI ratio in αβ and γδ splenocytes. **c**) The percentage of $GFP^+$ in gated $CD4^+$ γδ thymocytes from *Nfatc1-Egfp* mice. Left, the percentage of $GFP^+$ in αβ vs γδ $CD4^+$ thymocytes from *Nfatc1-EGfp.* Right, GFP MFI ratio in αβ and γδ $CD4^+$ thymocytes. The MFI values are normalized with the $GFP^-$ cell populations**.** Data from at least three independent experiments are shown as mean ± SEM. The statistical significance was determined by unpaired student's t-tests. **p-value < 0.005, ***p-value < 0.001, ****p-value <0.0001, n.s. not significant.

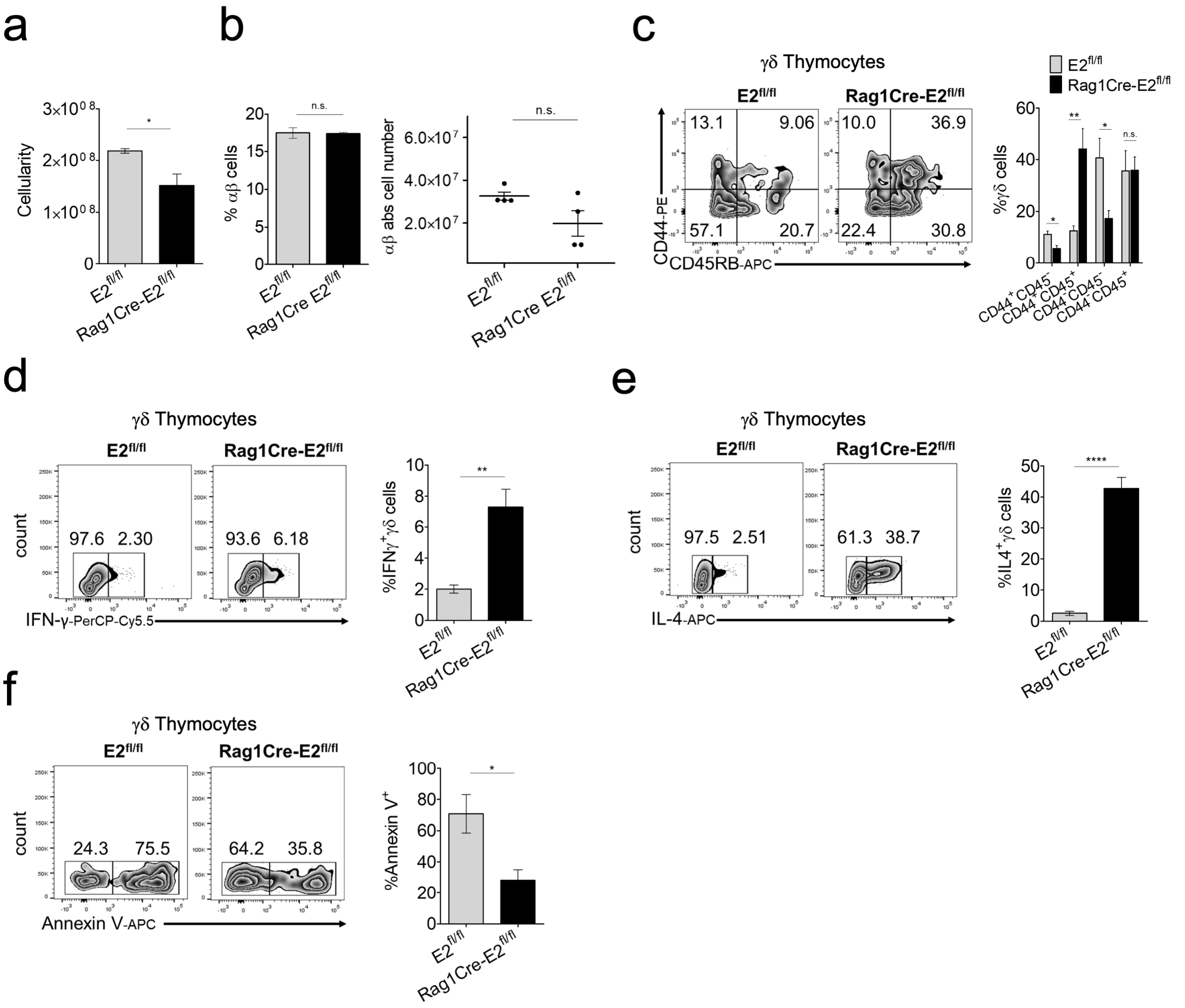


**Supplementary Figure 5. Deletion of remote E2 enhancer and, thereby, of NFATc1/α induction leads to a marked increase in NKT γδ cells. Related to Figure 5**

**a**) Thymic cellularity in the *E2fl/fl*, and *Rag1Cre-E2fl/fl* mice. **b**) Total thymocytes gated on living cells were stained with anti-TCRδ and anti-TCRβ (as in Figure 1d). Percentages (left) and absolute numbers of αβ cells (right) in the thymi of mice are shown. Each dot represents one mouse. **c**) Flow cytometry of *E2fl/fl* and *RagCre-E2fl/fl* γδ thymocytes stained with anti-CD44 and anti-CD45RB. Right, percentages of TCRγδ thymocytes expressing CD44 and CD45RB surface markers. **d**) IFN-γ and **e**) IL-4 production by *E2fl/fl* and *RagCre-E2fl/fl* γδ thymocytes. Right, the corresponding percentages of γδ thymocytes producing IFN-γ and IL-4 are shown. **f**) Flow cytometry of γδ thymocytes from *E2fl/fl* and *RagCre-E2fl/fl* mice upon annexin V staining. Right, percentages of annexin V$^+$ cells within γδ thymocyte population. Data from at least five independent experiments with three or four mice for each genotype are shown as mean ± SEM. The statistical significance was determined by unpaired student's t-tests. *p-value < 0.05, **p-value < 0.005, ****p-value <0.0001, n.s. not significant.

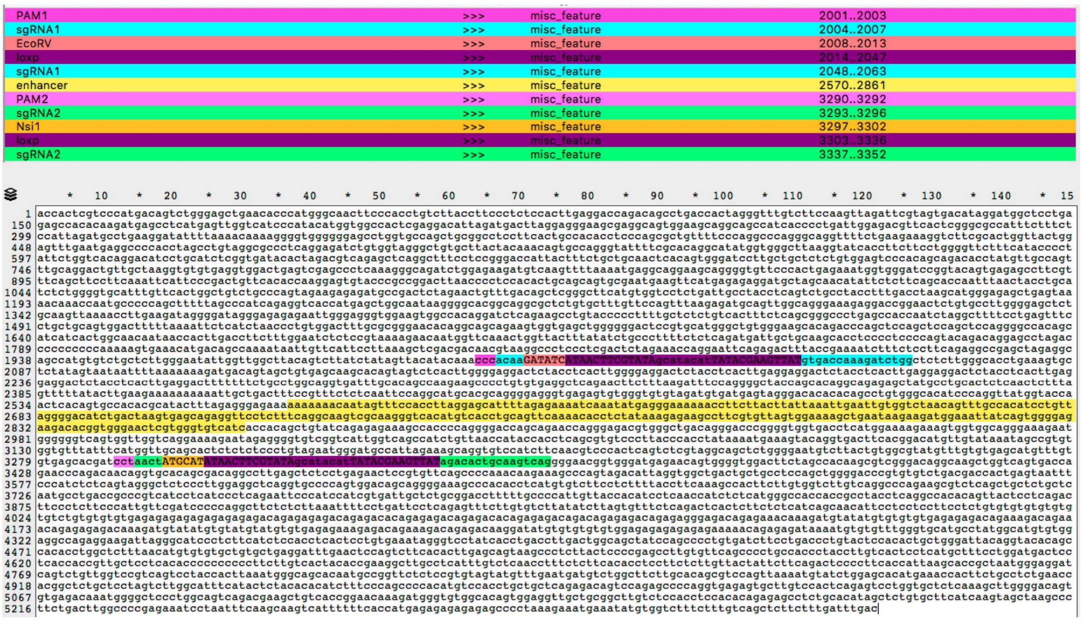


**Supplementary Figure 6. Targeting vector for E2$^{fl/fl}$. Related to STAR Methods**

Sequence and specific features of the targeting vector. An EcoRV and Nsi1 site was introduced next to the loxP sites to facilitate restriction enzyme diagnostic digests and the detection of correct targeting. The loxP sites are also positioned to disrupt the sgRNA binding site. This prevents the targeted allele from being cleaved by Cas9. ~2 kb of homology arms were used in the targeting vector. An enhancer sequence of the *Nfatc1* gene was chosen to be flanked by loxP sites.
